# The use of audio-visual aids to reduce delirium after cardiac surgery in intensive care units (DaCSi-ICU): A feasibility study protocol

**DOI:** 10.1371/journal.pone.0320935

**Published:** 2025-04-24

**Authors:** Maria Joao De Azinheira Reguenga, Smaragda Lampridou, Natalie Pattison, Stephen James Brett, Sanooj Soni

**Affiliations:** 1 Pre-Doctoral Research Fellow, Department of Surgery and Cancer, Imperial College London/Imperial College Healthcare NHS Trust,; 2 Doctoral Fellow, Department of Vascular Surgery, Imperial College London/Imperial College Healthcare NHS Trust,; 3 Professor of Clinical Nursing, University of Hertfordshire/East and North Herts NHS Trust & Researcher in Residence (Critical Care), Imperial College London/Imperial College Healthcare NHS Trust,; 4 Professor of Critical Care & Consultant in Intensive Care Medicine, Department of Surgery and Cancer, Imperial College London/Imperial College Healthcare NHS Trust,; 5 Clinical Senior Lecturer in Critical and Perioperative Care & Consultant in Intensive Care Medicine, Division of Anaesthetics Pain Medicine and Intensive Care, Department of Surgery and Cancer, Imperial College London/Imperial College Healthcare NHS Trust; Hospital Sirio-Libanes, BRAZIL

## Abstract

**Introduction:**

Delirium can affect over 50% of patients following cardiac surgery in intensive care units (ICU), leading to an increased risk of long-term cognitive impairment, prolonged hospital stays and increased costs. Nurse-led auditory-visual stimulation to help prevent and manage ICU delirium is a novel, unexplored strategy in postoperative cardiac surgical patients but proven to be effective in other long-term conditions. The Delirium after Cardiac Surgery in the Intensive Care Unit (DaCSi-ICU) study aims to assess the feasibility and acceptability of implementing an innovative, family-focused auditory-visual intervention to reduce delirium in ICU patients following major cardiac surgery.

**Methods and analysis:**

This is a pilot, mixed-methods, non-randomised feasibility study to be delivered in a university hospital cardiac ICU. The primary outcome is to explore the feasibility and acceptability of an innovative family-focused intervention to reduce ICU delirium rates in patients following cardiac surgery. Secondary outcomes are to: explore short-term post-surgical outcomes up to three months of hospital discharge; and investigate participants’ perspectives of taking part in the study. A total of 12 patients, alongside 12 family members or significant others and 6 ICU nurses will be recruited. Demographic data will be reported descriptively, and clinical data will be managed statistically through SPSS. Data collected from interviews will be transcribed full verbatim and analysed on NVIVO using framework analysis.

This study has received Health Research Authority (HRA) approval (24/YH/0011). Imperial College Healthcare NHS Trust is the sponsor for research governance purposes. This trial is registered at ClinicalTrials.gov (NCT06355570). Findings will be disseminated through peer-reviewed open-access journals and presented in national and international scientific meetings. Findings will also be shared with patients and the clinical team. Study results will determine the feasibility and acceptability of the intervention, facilitating the progression to a future controlled effectiveness trial.

## Introduction

Delirium is a type of confusion resulting from combined physiological, environmental and pharmacological factors that lead to brain dysfunction. This commonly occurs in intensive care units (ICUs) and is underdiagnosed, causing distress to patients, their family members and healthcare professionals [1,2]. It is associated with morbidity and increased 30-day mortality after hospital discharge by 1.3% compared to ICU delirium-free patients [3–5]. In critical care settings, the occurrence of delirium is reported to be around 30% according to meta-analyses of studies in ICU [6,7], which can increase to 60–80% depending on various risk factors and doubles the hospital inpatient cost per patient compared to those without delirium [8,9].

Patients undergoing cardiac surgery are a particularly vulnerable group and ICU delirium can affect over 50% of these patients due to a multitude of perioperative factors, including surgical complexity and prolonged cardiac bypass time [10–12]. Delirium post-cardiac operation is associated with increased anxiety and depression, poor functional status, increased risk of stroke and significant cognitive impairment up to a year post-cardiac surgery [10,11,13]. It has also been linked with an elevated number of hospital readmissions, longer ICU and hospital lengths of stay [10,11,14].

Given the significant burden on patients and the wider healthcare system, ICU delirium prevention and management was identified as a research priority in a James Lind Alliance research priority-setting partnership [14,15]. Management is principally supportive and various pharmacological interventions are the foundation of treatment, which can be ineffective [4,14,16]. However, the Society of Critical Care Medicine has highlighted the importance of non-pharmacological therapies [4] and there is an urgent unmet need to identify novel non-pharmacological management strategies to prevent and treat ICU delirium [4,15,17].

In recent years, family involvement has become a significant focus in the literature when debating patient outcomes in the healthcare system [4]. Several ICU studies have demonstrated positive results when involving family members in the management of ICU delirium [17–19]. For example, non-pharmacologic therapies involving family members led to a 24% risk reduction of delirium in a general ICU [20] whilst also reducing ICU delirium incidence compared to sedation reduction and exercise programmes [21,22]. Practically, family support is not available 24 hours a day in ICUs, especially during the night when ICU delirium can be more problematic [23]. To overcome this, an intriguing potential option is the use of family-focused sensory stimulation to provide patient re-orientation and reassurance [1], particularly in elective post-operative patients who are cognitively intact prior to the surgical procedure. However, it remains unknown whether audio-visual interventions combined with family involvement can prevent and manage ICU delirium [1,24,25], especially in the cardiac surgical field. Therefore, further studies are urgently required to investigate the use of family-centred sensory stimulation in ICU delirium prevention and management following cardiac surgery.

This protocol outlines procedures that will be conducted to answer the following research question: Is family-focused auditory-visual stimulation feasible and acceptable to be delivered in a critical care setting to patients’ post-cardiac surgery to reduce ICU delirium?

## Methods

This protocol report was developed in line with the Standard Protocol Items: Recommendations for Interventional Trials (SPIRIT) 2013 guidelines for pilot and feasibility trials, and the SPIRIT patient-reported outcomes (PRO) extension guidelines [26]. A detailed SPIRIT checklist for this study can be found in the S1 File. The trial was registered with ClinicalTrials.gov (NCT06355570).

### Study design

The DaCsi-ICU study is a feasibility pilot single-centre study and will implement an auditory-visual supportive package for patients recovering from major cardiac surgery at Imperial College Healthcare NHS Trust (ICHT), a tertiary university hospital group in West London, United Kingdom. This is a non-randomised study, and all study patients will receive the same intervention under evaluation. Study data collection will include gathering data through qualitative interviews, quality of life questionnaires (QoL) and patient’s medical records.

### Participants

This study involves the inclusion of patients, their families/friends and critical care nurses. Further details on participants’ eligibility criteria can be found in the S1 Table.

### Sample size

The study sample size was determined based on the complexity of the planned intervention and previous recommendations for pilot and feasibility studies [27]. Considering the study design and timelines, a total of 30 participants will be recruited for this research study by the Principal Investigator (PI). This will involve a recruitment target of 12 patients alongside 12 family members or close friends and 6 critical care nurses. This sample will allow the collection of sufficient in-depth data about the intervention’s acceptability in the ICU, and the feasibility of a future randomised study.

### Recruitment

Patients will be recruited considering their priority for surgery and eligibility for the study and will be identified by the PI and the clinical care team at the pre-operative assessment clinic in Hammersmith Hospital (HH). The PI will remotely screen for potential participants using the following sources: pre-operative cardiac clinic lists, cardiothoracic theatre lists at HH and weekly multidisciplinary team meetings. Subsequently, the PI will consult patient medical electronic records to confirm their suitability and eligibility for the study.

Patients and family members/friends will be approached face-to-face, screened and recruited at the ICHT pre-operative assessment clinic outpatient department at HH. Participants will be given the patient information sheet (PIS) in person and sufficient time, expected to be at least 24 hours to consider their participation in the research study (S2 File). Alternatively, potential participants may be contacted via telephone and introduced to the study after being initially approached by the clinical team at the pre-operative assessment. Those wishing to take part will be sent an information sheet and informed consent form via email or in the post.

Patient participants will be asked to nominate a significant other and if they would be willing to give a PIS to their chosen person. Significant others will be given time to consider their participation and if content, they will be asked to sign a consent form after the study PI has clarified all questions (S3 File).

In addition, patients will be asked to select a personal consultee to be involved in situations where they might lack the capacity to verbally consent to the study (e.g., delirium episodes). Personal consultees can be anyone within the patient’s family or friend’s group (e.g. relative/friend/partner) and will be required to sign an information sheet after considering their role in the study (S4 File) [28].

Six critical care nurse participants will be identified in the ICU after providing direct care to at least one participant enrolled in the research study. They will be invited to participate and asked to sign an informed consent form (S5 File).

### Study procedures

**Fig 1:**
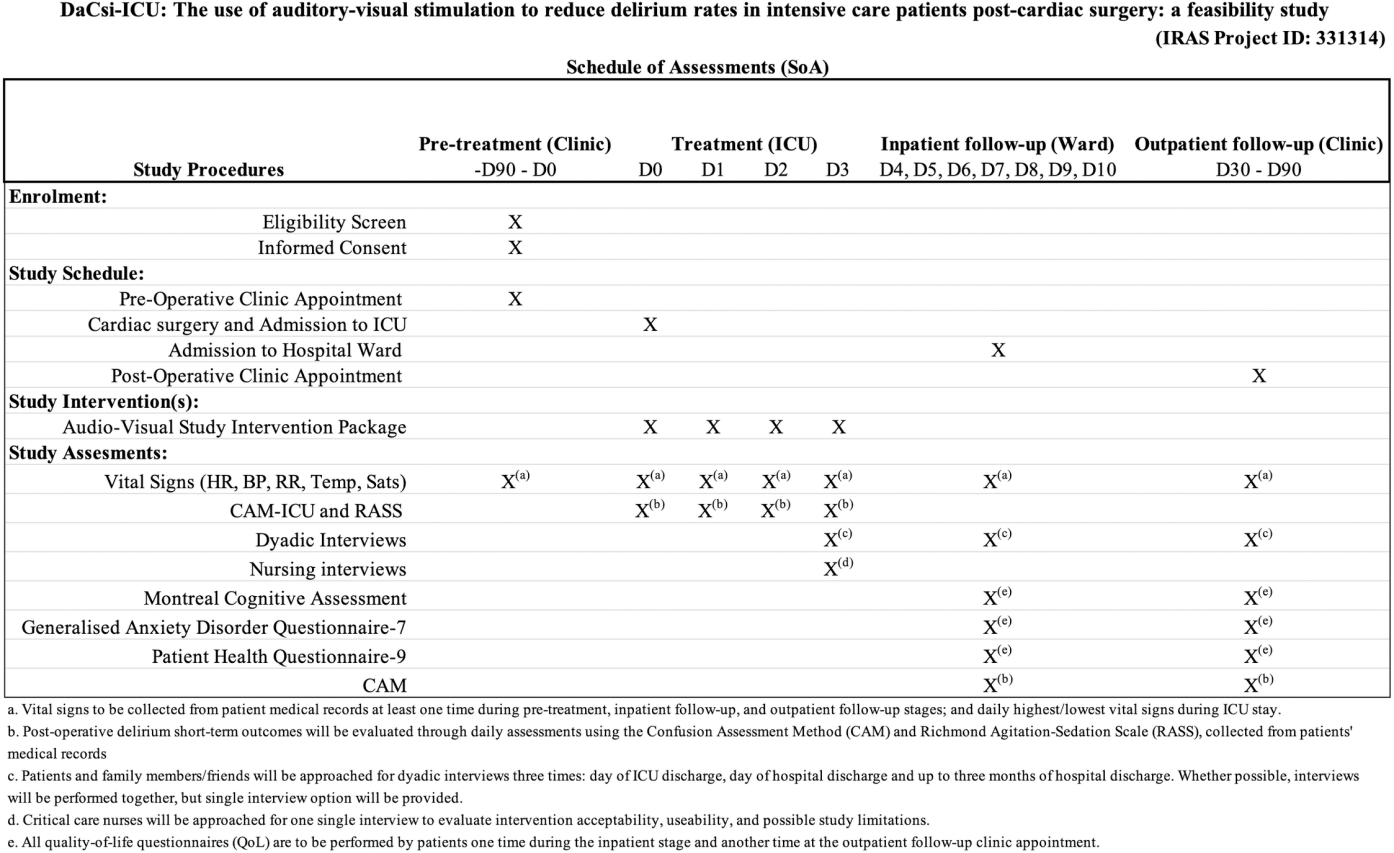
Study Schedule of Assessments.

Various data will be collected at the different study timelines and study participants will undergo the schedule of assessments (SoA, Fig 1).

**Fig 2:**
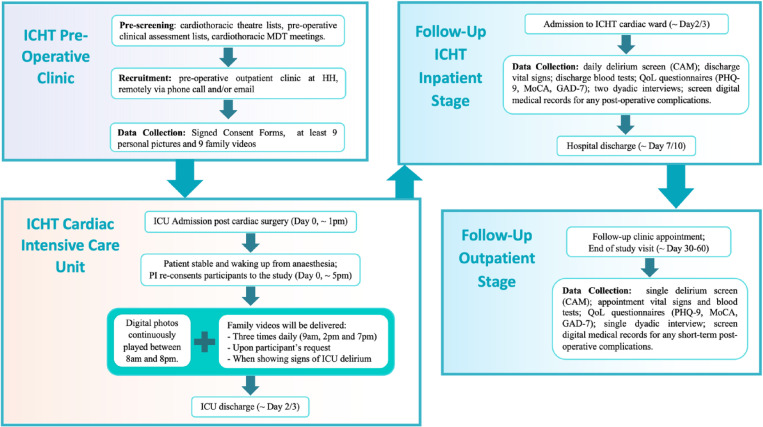
Patient pathway flowchart.

This study comprises four different phases: 1) Pre-operative, 2) ICU, 3) Inpatient hospital follow-up and 4) Outpatient clinic follow-up (systematically summarised in the study patient pathway flowchart – Fig 2).

### Study intervention

The study intervention will require the recording of videos and the selection of personally significant pictures. The PI will be responsible for setting up the required equipment and delivering the intervention in the immediate post-operative stage alongside ICU nurses. The Template for Intervention Description and Replication (TIDieR) checklist has been used to systematically present the study intervention and its components in the S2 Table [29].

To ensure the correct delivery of the study intervention in the immediate post-operative phase, the recording of videos by significant others and the selection of personal pictures by patients will take place during the preoperative stage. The selection of pictures will be based on significant things to the patients. Videos will follow a script divided into two parts: a) Reorientation (discussing date/time/surroundings, and family members on the video) and, b) Reassurance (providing comforting/supportive messages).

The suggested guided video script will be provided to family members or friends at the pre-operative assessment clinic. This guide was initially developed based on published research and considering previous patients’ memories from their lived ICU experiences [22]. Subsequently, it was further refined within a lay focus group of patients and public members (with ICU lived experience) and reviewed by an expert panel comprising two critical care consultants, two critical care nurses and one clinical academic research nurse. The final version of the agreed video script can be found in S6 File.

### Outcome measures

Primary and secondary outcome measures are detailed in the S3 Table. Any other relevant measures that will be collected during the study can be found in the S4 Table.

Considering the novelty of the proposed study intervention and the lack of similar interventional trials performed within the specific target population (e.g., post-cardiac surgery), evaluating the practicalities and acceptability of the study procedures is paramount to establish the need for further refinements. This will contribute towards the progression of this study to a randomised controlled trial, allowing a comprehensive evaluation of the effectiveness of the study intervention [30]. Determining the intervention’s feasibility and acceptability will contribute to the decision of whether to proceed to a full randomised controlled trial (RCT), allowing a comprehensive evaluation of the effectiveness of the study intervention. Upon the DaCSi-ICU study completion, the study team will meet and reach a consensus on whether the study is: a) ready to proceed to an RCT; b) ready pending some actions/improvements; and c) not ready to proceed to an RCT [31]. In addition, the study team has agreed on explicit metrics that will contribute to the decision of whether to proceed to the next stage of the research [31–33]:

a) An estimated recruitment rate of 1.5 per month (which translates into successful recruitment of 6 patients in the first 4 months)
b) A refusal rate of ≥ 40% of participants will lead to a protocol revision
c) A retention rate of 100% of those recruited by the inpatient follow-up for purposes of achieving the primary outcomes (feasibility and acceptability of the study intervention).
d) A retention rate of ≥ 75% of those recruited to provide outpatients follow-up measures (3 months), including secondary outcome measures.
e) A good intervention adherence: ≥ 70% of patients were administered the study interventions per protocol (data will be retrieved from the content intervention monitoring checklist – S7 File).

### Interviews

Study participants will participate in qualitative interviews, which are anticipated to run for approximately 30 minutes. Dyadic interviews will be undertaken with patients and family members to better comprehend patients’ experiences and the impact of the intervention on both the patient and significant others [34]. One-to-one interviews with healthcare professionals will also enable a deeper exploration of the intervention’s implementation, challenges, and opportunities for improvement. Interview topic guides were carefully designed within a group of two ICU consultants, two ICU nurses, and one clinical academic fellow, two of whom are experts in qualitative research. The topic guides were refined following pilot testing within a small patient and public involvement group of five members (three patients and two family members with previous lived ICU experience).

Patients and significant others will take part in three distinct dyadic interviews. The first two will take place during inpatient stay time to explore their acceptance of the study intervention and ICU experience. If this is not feasible, the last interview will be conducted via telephone in the immediate days following discharge. The third interview will take place in person, at the post-operative follow-up appointment, or virtually, depending on participants’ preference, at around three months after hospital discharge, to explore secondary study outcomes.

Where possible, interviews with family members/friends and patients will be conducted together, but the possibility of performing interviews separately will be available based on the participants’ preferences. Interviews with nurses will take place either virtually (by phone or video call) or in private rooms located in the ICU and will aim to investigate the feasibility and acceptability of implementing the study intervention in the ICU.

## Data management and analysis

### Data collection and management

Following the recruitment and consideration of each patient’s preference, the recording of family videos and transfer of digital pictures will be completed on the same day as consenting, or during any other hospital appointment arranged within the pre-operative stage. Alternatively, patients and family members will be given the possibility to transfer the data via email to the PI’s secure NHS email any time before surgery.

In the ICU, the research team will mostly administer the study intervention (personal photos and family videos). In occasions where the research team is not available, ICU nurses take part in the delivery and they will be trained beforehand. Irrespective of this, all critical care nurses will contribute to the study by filling in a daily checklist as part of data collection (S7 File), which will help the research team understand the impact of the study intervention on patients. Practical aspects, including placing visual/hearing aids (e.g. earplugs/limited vision) before delivering the study intervention, have been highlighted in this checklist.

Whilst patients remain in the hospital, clinical data will be collected from medical records to assess various outcome measures. Delirium assessments will be prospectively carried out every 12-hour shift by ICU nurses using the Confusion Assessment Method for the ICU (CAM-ICU) tool, which will be documented in the patient’s medical records as per hospital policy. These CAM-ICU scores will be collected by the research team (S4 Table). Following ICU discharge, each patient will be followed up daily on the ward and once after hospital discharge at the standard of care monthly follow-up clinic. During this stage, QoL questionnaires, including cognitive and emotional assessments, will be administered as the international community recommends when evaluating delirium outcomes in ICU survivors [35]. Cognitive and emotional evaluations will be performed according to the schedule of assessment and will include the use of the following tools: CAM, MoCA (Version 8.3), GAD-7 and PhQ-9 [36–38].

Interviews will be audio recorded with an encrypted device provided by the research team and transcribed full-verbatim by an approved third-party transcription company, who will subsequently remove any identifiable data from transcripts. All interviews will be carried out by the same researcher (PI) throughout the study to reduce differences in the way questions are asked.

Videos will be recorded either with a video-recorded device that belongs to the study team or self-recorded by family members or friends at home using a personal filming device (e.g., smartphone). Subsequently, videos and pictures will be sent to the PI via the NHS Egress system and stored on a secure institutional password-protected computer, before being transferred to the study digital equipment. All video recordings and personal pictures will be deleted after the study intervention is finished or upon ICU discharge. No videos and/or photos will be retained by the study team without seeking participants’ prior consent.

The PI will be taking the lead on the research study and will be responsible for collecting, managing, and analysing the trial data. Qualitative data management and outcomes will be discussed with the two co-investigators, considering their level of expertise in the field. Any other generated trial data and quantitative outcomes will be reported to the study’s Chief Investigator (CI) and, subsequently, discussed amongst the wider study team.

### Data analysis

Due to the study’s small sample size, both qualitative and quantitative findings will be analysed and reported with careful consideration of their limitations.

#### Quantitative analysis.

Analysing study feasibility and acceptability will involve screening the study database and daily nursing checklists to evaluate the study process metrics, which include:

a) Study reach (proportion of eligible patients who were offered and received the intervention)
b) Intervention fidelity and consistency (the extent to which the intervention was delivered as intended and sustainability)
c) Resources used (staff time, equipment, etc.)
d) Patient adherence to the protocol (compliance with the study intervention).

The study database will be screened to establish recruitment, uptake and dropout rates, patient willingness to take part in the study and the ability to retain participants. These measures will contribute to better comprehending future recruitment barriers and/or facilitators factors, calculating the required sample size for a future trial, and leading to a more effective and cost-effective study design.

Secondary outcomes of the study will be assessed based on the clinical characteristics of patients during their ICU stay and patient-reported outcomes from both interviews and medical records.

Additionally, other relevant measures will be analysed using IBM Statistical Package for the Social Sciences (SPSS) statistical software version 29. Descriptive analyses will be performed to characterise the study population using:

- Frequency distributions (e.g., ICU delirium outcome)
- Measures of central tendency (e.g., mean age)
- Measures of dispersion (e.g., standard deviation for age)
- Cross-tabulations to present proportions and the distribution of relevant predictors (e.g., comorbidities, socio-demographic factors), as well as study secondary outcomes – contingency tables will be created to explore potential associations between the development of delirium and one or more variables.

#### Qualitative analysis.

Analysis of the study’s feasibility and acceptability will include conducting semi-structured interviews to explore participants’ perceptions of the study intervention benefits, appropriateness, and overall experience.

Data will be systematically managed and analysed in Lumivero NVIVO software version 14. A combined framework approach will be employed to systematically explore and categorise participants’ perceptions of the practicalities and suitability of the study intervention. This approach enables themes to be developed both inductively (from participants’ reported experiences) and deductively (from existing literature) providing a comprehensive interpretation of the data [39].

The PI will compare the interview recordings with transcripts to address any inconsistencies before analysis. This process will enhance familiarisation with the data, alongside re-reading and listening to interview transcripts, which will be essential to highlighting key participants’ quotes and fundamental to identifying initial patterns in the data.

Charting (via line by line) and indexing will be used to develop the coding frame, as per framework analysis approaches [39]. This process will involve assigning labels to key concepts, noticing any similarities across transcripts and single coding performed by the PI on the entire dataset. Following the development of a coding tree, codes will be organised and grouped into categories based on their context. Initial themes will be developed considering the emerged categories and their alignment with the study objectives.

Following the identification of initial themes, the PI will review the data and revisit the coding frame (iterative process) to ensure selected themes reflect data collected from interviews [39]. Emerging themes within the framework will be then discussed with two co-investigators, who are qualitative experts and critical care physicians. In addition, to address issues of confirmability and dependability, the PI will compare identified themes with existing literature [40]. Moreover, to enhance transferability the lead researcher will also liaise with a patient and public involvement (PPI) advisory group to check the credibility and usefulness of the findings.

Trustworthiness of the data will be managed by supplementing transcripts with field notes taken during or immediately after interviews [40]. In addition, the PI will maintain a reflexive journal to document any challenges met during interviews or modifications to the topic guide, also addressing credibility. Moreover, the lead researcher will engage in regular discussions with co-investigators and regularly update the reflexive journal on any thoughts and emotions felt during interviews.

To ensure rigour throughout data collection and analysis, the PI will also maintain a documentation log detailing any decisions taken during the trial and reflections on how any biases were addressed, as well as, ensure transparency on documentation and management of the research study.

### Ethical approval

The study coordination centre obtained favourable approval from the Bradford and Leeds Research Ethics Committee (REC) and Health Research Authority (HRA) in January 2024, with reference number 24/YH/0011. The study also received NHS Sponsorship and Research Information Governance approval in February 2024, together with a Confirmation of Capacity and Capability to conduct the study at ICHT (23HH8130). All approvals were received before any research activity was carried out. Recruitment activities began in March 2024 and the first study participant was successfully enrolled to the study on the 6^th^ of April 2024.

### Consent

Consent to enter the study will be sought from each study participant only after a full explanation has been given, an information leaflet offered, and time allowed for consideration and questions. A signed participant consent will be obtained before their enrolment into the study and consent will be dealt with as an ongoing process before any study task takes place. The right to refuse to participate without giving reasons will be respected by the research team. All participants will be free to withdraw at any time from the study and they will be made aware that their participation in the study will not affect their care or employment in any way. The research team will also ensure that participants’ wishes are documented on medical notes and the clinical team is aware of the study team’s plan of action.

Due to the specificity of the care group, the patient’s mental capacity is expected to fluctuate throughout the study. The research team will continuously assess patients’ capacity and ability to re-consent to the study. The study intervention will commence once verbal consent is obtained from patients after they have awoken from deep sedation. In addition, a Patient Regaining Capacity Consent form will be signed post-operatively and, ideally, before patients resume the study in the ICU or after any periods of lacking capacity (e.g., surgery). In situations of delirium, a personal consultee will be contacted and enquired about the patient’s wishes to participate in the study.

### Harms/ indemnity

Due to the nature of the study design, we do not expect any adverse or serious adverse events. If a participant should experience an event of clinical interest whilst in the hospital, standard NHS pathways will be followed. If any incidental findings and/or other patient safety concerns are identified by the research team, this will be immediately reported to the CI, and appropriate support measures will be implemented. In these situations, documentation will be kept in the patient’s medical records and filed in the study site file.

Participation in the study might trigger experiences that patients and significant others find difficult to manage. For instance, during interviews, participants may find it upsetting when discussing personal experiences, if so, they will be free to pause or stop at any point in an interview. In situations of distress, additional support (e.g., counselling) will be offered and organised as per standard NHS care. Alternatively, a referral will be made to the intensive care multi-professional follow-up care team, who have direct access to NHS psychologist services.

The Sponsor holds a standard NHS hospital indemnity and insurance cover with NHS Resolution for NHS Trusts in England, which applies to this study. As part of the study information sheet, participants will also be given written advice and appropriate contact details (e.g., Chief and Principal Investigators, Patient Advice and Liaison Service) to escalate any concerns or complaints that they might have during the study.

### Protocol amendments/deviations

Every care was taken when drafting the study protocol (S8 File), but corrections or amendments may be necessary in the future. These will be circulated to investigators in the study before re-submission. Problems relating to this study should be referred, in the first instance, to the CI.

No amendments to this protocol will be made without prior consultation and agreement with the Sponsor. Any amendments to the study that appear necessary during the study must be discussed with the Research Team and the Sponsor concurrently. If an agreement is reached concerning the need for an amendment, it will be produced in writing by the PI and will be made a formal part of the protocol following ethical and regulatory approval.

Any protocol deviations will be documented in a protocol deviation form and safely filed in the Trial Master File. The CI and co-investigators will be made aware of the protocol deviation. Corrective actions and preventative actions will be discussed and implemented to prevent new deviations from the study protocol.

### Dissemination policy

The PI will coordinate the dissemination of data from this study and ensure all published/presented data will be unidentifiable. All publications (e.g., manuscripts, abstracts, oral/slide presentations, book chapters) based on this study will be reviewed by each co-investigator before submission. Any published interview data will be anonymised, including direct quotations from participants that were essential to illustrate key themes. Communication concerning the study, including scientific meetings, will acknowledge the responsible parties and the invaluable Imperial Health Charity’s financial contribution and continuous support to this research study.

We intend to disseminate the study findings via peer-reviewed open-access journals and conduct oral presentations at both national and international research meetings within the critical care field. Moreover, results will be shared within a patient and public involvement focus group involved in the co-design of the study intervention and with study participants upon written request. Study insights will also be communicated within local research-led meetings and local cardiac ICUs.

## Supporting information

S1 Table Study Eligibility Criteria. (DOCX)

S2 Table TiDIER Study Intervention. (DOCX)

S3 Table Study outcomes related to the feasibility and acceptability of the intervention. (DOCX)

S4 Table Other outcomes and relevant measures for cohort description. (DOCX)

S1 File SPIRIT 2013 Checklist for the DaCSi-ICU Study. (DOCX)

S2 File PIS for Patients. (PDF)

S3 File PIS for Family Members or Friends. (PDF)

S4 File Information Sheet for Personal Consultees. (PDF)

S5 File PIS for Critical Care Nurses. (PDF)

S6 File Video Script for Family Members/Friends. (DOCX)

S7 File Study Daily Checklist. (DOCX)

S8 File Study Protocol v1.3 01Dec2024. (PDF)
