# Supplementary material for: The use of audio-visual aids to reduce delirium after cardiac surgery in intensive care units (DaCSi-ICU): A feasibility study protocol
Source: PLoS One. 2025 Apr 24;20(4):e0320935. doi: 10.1371/journal.pone.0320935 (PMC12021270; doi:10.1371/journal.pone.0320935)
Supplement: S1 Table — (DOCX) [file pone.0320935.s001.docx]

**S1 Table. Study Eligibility Criteria**

Table 1. Study Eligibility Criteria

| Participants | *Eligibility Criteria* |
| --- | --- |
| Patients | 1) over the age of 18;  2) able to speak the English language;  3) have the mental capacity to consent to the study pre-operatively;  4) scheduled to undergo cardiac surgery at ICHT;  5) do not have any significant visual and hearing impairment;  6) willing to provide personal pictures;  7) able to select a family member or friend to participate in the research study;  8) do not have pre-existing delirium, dementia or other significant underlying cognitive impairment;  9) unlikely to die within 24 hours following cardiac operation. |
| Family members or friends (termed significant others) | 1) nominated by patients undergoing a cardiac operation at ICHT;  2) able to speak the English language;  3) willing to record videos;  4) have the mental capacity to consent to the study pre-operatively;  5) do not have any significant visual and hearing impairment. |
| Critical care nurses | 1) having provided direct nursing care to at least one study participant;  2) willing to provide an audio-recorded interview. |
