# Supplementary material for: The use of audio-visual aids to reduce delirium after cardiac surgery in intensive care units (DaCSi-ICU): A feasibility study protocol
Source: PLoS One. 2025 Apr 24;20(4):e0320935. doi: 10.1371/journal.pone.0320935 (PMC12021270; doi:10.1371/journal.pone.0320935)
Supplement: S2 Table — (DOCX) [file pone.0320935.s002.docx]

**S2 Table. TiDIER Study Intervention**

Table 2. TiDIER Study Intervention

| N. | Item | Description |
| --- | --- | --- |
| 1 | Brief Name | Nursing-led family centred audio-visual sensory aids |
| 2 | Why (rationale) | Addressing ICU delirium can be challenging, and current management options for this condition are limited. This study will investigate a novel, non-pharmacological intervention focused on family involvement, which has the potential to improve patient outcomes, enhance their recovery, and reduce the burden of delirium on patients and ICU settings. |
| 3 | What (materials) | The study intervention is a combination package that requires the collection of at least:  - 10 personal pictures  - 9 family videos (no longer than 3-minutes each) |
| 4 | What (procedures) | - Personal pictures will be selected by patients  - Short-videos will be recorded by significant others and based on a guided script (Appendix III) |
| 5 | Who (provided) | PI alongside ICU nurses will deliver the study intervention |
| 6 | How (delivery) | - Pictures to be displayed on a digital photo frame  - Videos will be played on an iPad/Tablet device |
| 7 | Where (location) | Intervention will be only delivered during patient stay in ICU (Flowchart 1) – in secondary care |
| 8 | When (frequency) | - Pictures to be continuously shown between 8am to 8pm  - Videos to be played at specific times: 9am, 2pm and 7pm  - Videos to be re-played in addition to the planned set times upon patient request and/or when patients develop ICU delirium. |
| 9 | Tailoring | Participants’ wishes will be respected when altering any part of the study intervention. |
| 10 | Modification | Study intervention has been carefully designed, but any modifications will be considered after analysing the study. Modifications to the protocol will be reported in the feasibility study paper. |
| 11 | How well (plan) | - ICU Nurses will be trained on how to use the digital equipment and deliver study intervention to ensure consistency;  - A flag trial sheet will be placed at patient bedside to immediately highlight patients participating in the research study;  - An instructive research note will be added to participant’s medical records;  - The PI will shadow the study intervention deliver by nurses to ensure adherence to the protocol;  - Participant engagement to the intervention will be recorded in the Study Daily Checklist (Appendix II);  - Any deviations will be recorded and preventative actions implemented. |
| 12 | How well (actual) | To be assessed |
