# Supplementary material for: The use of audio-visual aids to reduce delirium after cardiac surgery in intensive care units (DaCSi-ICU): A feasibility study protocol
Source: PLoS One. 2025 Apr 24;20(4):e0320935. doi: 10.1371/journal.pone.0320935 (PMC12021270; doi:10.1371/journal.pone.0320935)
Supplement: S3 Table — (DOCX) [file pone.0320935.s003.docx]

**S3 Table. Study outcomes related to the feasibility and acceptability of the intervention**

Table 3. Study outcomes related to the feasibility and acceptability of the intervention

| **Outcome Measures** | **Data Source** |
| --- | --- |
| Assessing Study Intervention Feasibility and Acceptability | |
| Experience of patients receiving the intervention | Qualitative Interviews |
| Experience of significant others preparing the intervention | Qualitative Interviews |
| Experience of critical care nurses delivering the intervention | Qualitative Interviews |
| Compare number of patients willing to those unwilling to receive the intervention | Study database |
| Determine barriers and/or facilitator factors of implementing a supportive programme and the potential impact on patients, family members and critical care staff | Daily Checklists & Qualitative Interviews |
| Explore compliance rates and patient adherence to the study intervention | Daily Checklists |
| Evaluate Study Recruitment and Retention Rates | |
| Recruitment proportion (e.g., number of participants interested in and eligible to receive the intervention) | Study database |
| Uptake proportion (e.g., number of eligible patients approached and consented to the study) | Study database |
| Dropout/Retention proportion (e.g., number of participants wishing to withdraw from the study) | Study database |
| Number of patients who withdraw or have missing data | Study database |
| Study Secondary Outcomes | |
| Explore participants’ ICU experience and reflections on study participation. | Qualitative Interviews |
| Explore short-term post-surgical outcomes up to three months of hospital discharge. | Qualitative Interviews & Questionnaires |
