# Supplementary material for: The use of audio-visual aids to reduce delirium after cardiac surgery in intensive care units (DaCSi-ICU): A feasibility study protocol
Source: PLoS One. 2025 Apr 24;20(4):e0320935. doi: 10.1371/journal.pone.0320935 (PMC12021270; doi:10.1371/journal.pone.0320935)
Supplement: S4 Table — (DOCX) [file pone.0320935.s004.docx]

**S4 Table. Other outcomes and relevant measures for cohort description**

Table 4. Other outcomes and relevant measures for cohort description

| **Domain** | **Measures** | **Collection Timeline** |
| --- | --- | --- |
| Socio-Demographic Details* | Patient’s age, gender, ethnicity | Pre-Operative |
| Clinical Factors* | Past medical history, baseline vital signs measurement (heart rate, blood pressure, respiratory rate, oxygen saturation levels and temperature), concomitant medication at home, baseline weight and height | Pre-Operative |
|  | Cardio-pulmonary bypass time, cross-clamp duration and any blood products transfusions administered | Intra-operative |
|  | Every 12-hour shift vital signs measurements, neurological assessments (through Richmond Agitation Sedation Scale and Confusion Assessment Method (CAM – ICU)).  Daily blood tests (including electrolytes, renal function, liver function tests, bone profile and full blood count), drug chart (including infusions and daily doses administered), mechanical ventilation duration. | Critical Care |
|  | Daily CAM assessments, discharge vital signs and blood tests | Inpatient and Outpatient Follow-up |
| Psychosocial Factors | Patient Health Questionnaire (PHQ-9) | Inpatient and Outpatient Follow-up |
|  | Montreal Cognitive Assessment (MoCA) questionnaire |  |
|  | Generalized Anxiety Disorder 7 (GAD-7) questionnaire |  |

**Data will be retrospectively collected from patients’ digital medical records.*
