# Supplementary material for: The use of audio-visual aids to reduce delirium after cardiac surgery in intensive care units (DaCSi-ICU): A feasibility study protocol
Source: PLoS One. 2025 Apr 24;20(4):e0320935. doi: 10.1371/journal.pone.0320935 (PMC12021270; doi:10.1371/journal.pone.0320935)
Supplement: S1 File — (DOCX) [file pone.0320935.s005.docx]

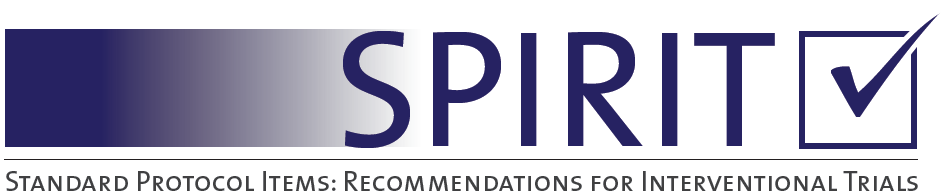


**S1 File. SPIRIT 2013 Checklist for the DaCSi-ICU Study**

| Section/item | ItemNo | Description |
| --- | --- | --- |
| **Administrative information** | | |
| Title | 1 | The use of audio-visual aids to reduce Delirium after Cardiac Surgery in Intensive Care Units (DaCSi-ICU): A Feasibility Study Protocol |
| Trial registration | 2a | ClinicalTrials.gov (NCT06355570) |
|  | 2b | WHO Trial Registration Data Set:  1. Primary Registry and Trial Identifying Number:   - IRAS Project ID: 331314 - REC reference: 24/YH/0011   2. Date of Registration in Primary Registry: 30^th^ January 2024  3. Secondary Identifying Numbers:   - Sponsor: 23HH8130 - Funders: RFPR2324_9   4. Source(s) of Monetary or Material Support: Awarded Imperial Health Charity Grant (Pre-Doctoral Research Fellowship)  5. Primary Sponsor: Imperial College Healthcare NHS Trust (ICHT)  6. Secondary Sponsor(s): N/A  7. Contact for Public / 8. Scientific Queries:   - Study Coordinator: Maria Reguenga ([maria.reguenga@nhs.net](mailto:maria.reguenga@nhs.net))   9. Public/Small Title: Delirium after Cardiac Surgery in Intensive Care Units (DaCSi-ICU)  10. Scientific/Full Title: The use of audio-visual aids to reduce Delirium after Cardiac Surgery in Intensive Care Units (DaCSi-ICU): A Feasibility Study Protocol  11. Countries of Recruitment: United Kingdom  12. Health Condition(s) or Problem(s) Studied: Delirium in Critical Care Units  13. Intervention(s): Please refer to section 11a of this checklist.  14. Key Inclusion and Exclusion Criteria: Please refer to section 10 of this checklist.  15. Study Type: Please refer to section 8 of this checklist.  16. Date of First Enrolment: Actual Date: April 2024  17. Sample Size: Please refer to section 14 of this checklist  18. Recruitment Status: Suspended: there is a temporary halt in recruitment and enrolment (the recruitment target has been met, but not all participants have completed the study yet).  19. Primary Outcome(s) / 20. Key Secondary Outcomes: Please refer to section 12 of this checklist.  21. Ethics Review: Please refer to section 24 of this checklist.  22. Completion date: Expected by March 2025  23. Summary Results: Pending (trial data not analysed, thus study results not yet generated)  24. IPD sharing statement: Please refer to section 31a and 31b of this checklist. |
| Protocol version | 3 | Study Protocol Version 1.3 and Dated 01/Dec/2023 |
| Funding | 4 | Funding was provided by Imperial Health Charity (IHC) towards the lead researcher salary costs and other research costs (e.g., equipment, stationery items, etc.) |
| Roles and responsibilities | 5a | - Maria Reguenga, Pre-Doctoral Research Fellow, Department of Surgery and Cancer, Imperial College London/Imperial College Hospitals NHS Trust (maria.reguenga@nhs.net)  - Smaragda Lampridou, Doctoral Fellow, Department of Vascular Surgery, Imperial College London/Imperial College Hospitals NHS Trust (s.lampridou@nhs.net)  - Natalie Pattison, Professor of Clinical Nursing, University of Hertfordshire/East and North Herts NHS Trust & Researcher in Residence (Critical Care), Imperial College London/Imperial College Hospitals NHS Trust (n.pattison@herts.ac.uk)  - Stephen J. Brett, Professor of Critical Care & Consultant in Intensive Care Medicine, Department of Surgery and Cancer, Imperial College London/Imperial College Hospitals NHS Trust ([stephen.brett@imperial.ac.uk](mailto:stephen.brett@imperial.ac.uk))  - Sanooj Soni, Clinical Senior Lecturer in Critical and Perioperative Care & Consultant in Intensive Care Medicine, Division of Anaesthetics Pain Medicine and Intensive Care, Department of Surgery and Cancer, Imperial College London/Imperial College Hospitals NHS Trust (sanooj.soni@nhs.net) |
|  | 5b | Dr Rinat Ezra, Clinical Trials Manager, Research Governance and Integrity Team (RGIT), Imperial College London/Imperial College Healthcare NHS Trust (r.ezra@imperial.ac.uk) |
|  | 5c | Please see 21b |
|  | 5d | Please see 21a |
| Introduction |  |  |
| Background and rationale | 6a | Research Question: Is family-focused auditory-visual stimulation feasible and acceptable to be delivered in a critical care setting to patients’ post-cardiac surgery to reduce ICU delirium?  Justification for the trial: Delirium prevention and management in critical care units is a national priority and current treatment options are often ineffective or sub-optimal. Hence, innovative strategies are needed to effectively manage delirium in ICUs. |
|  | 6b | N/A |
| Objectives | 7 | We hypothesise that the use of auditory and visual sensory stimulation combined with family involvement/interventions can potentially improve delirium outcomes in critical care units following cardiac surgery.  (Please refer to Table 3 for further details). |
| Trial design | 8 | This is a pilot, mixed-methods, non-randomised feasibility study |
| Methods: Participants, interventions, and outcomes | | |
| Study setting | 9 | Single centre university hospital cardiac surgical ICU |
| Eligibility criteria | 10 | Patients: 1) over the age of 18; 2) able to speak the English language; 3) have the mental capacity to consent to the study pre-operatively; 4) scheduled to undergo cardiac surgery at ICHT; 5) do not have any significant visual and hearing impairment; 6) willing to provide personal pictures; 7) able to select a family member or friend to participate in the research study; 8) do not have pre-existing delirium, dementia or other significant underlying cognitive impairment; 9) unlikely to die within 24 hours following cardiac operation.  Significant Others: 1) nominated by patients undergoing a cardiac operation at ICHT; 2) able to speak the English language; 3) willing to record videos; 4) have the mental capacity to consent to the study pre-operatively; 5) do not have any significant visual and hearing impairment.  Critical Care Nurses: 1) having provided direct nursing care to at least one study participant; 2) willing to provide an audio-recorded interview. |
| Interventions | 11a | The study intervention involves a combination package that requires at least:  - 10 personal pictures to be continuously shown between 8am to 8pm  - 9 family videos* (no longer than 3-minutes each) to be played at specific times: 9am, 2pm and 7pm  *Videos to be re-played in addition to the planned timepoints upon request and/or when patients develop ICU delirium.  (Please refer to the TiDIER Checklist for further details). |
|  | 11b | Participants Withdrawal Criteria:  - No longer willing to participate after consent  - Personal consultee wishing to withdraw participants from the study  - Patients that the clinical and/or research team deem too distressed to continue participating in the study  - Participants who have lost capacity to participate in the study after hospital discharge |
|  | 11c | 1. Nurses will be trained on how to use the digital equipment and deliver study intervention to ensure consistency.  2. A flag trial sheet will be placed at patient bedside to immediately highlight patients participating in the research study.  3. An instructive research note will be added to participant’s medical records.  4. The PI will shadow the study intervention deliver by nurses to ensure adherence to the protocol.  5. Participant engagement to the intervention will be recorded in the Study Daily Checklist.  6. Any deviations will be recorded, and preventative actions implemented. |
|  | 11d | Whilst patients remain in hospital, clinical data will be collected from medical records to assess various outcome measures.  (Please refer to Table 4 for further details). |
| Outcomes | 12 | Primary outcomes:  - Assess the feasibility of implementing a family-focused sensory stimulation to improve ICU delirium outcomes in patients undergoing cardiac surgery.  - Explore the acceptability of an innovative programme to be implemented in cardiac surgical ICU, amongst critical care staff, patients and family members/friends.  Secondary outcomes:  - Investigate patients’ and family members’ perspectives of ICU experience and their reflections on study participation.  - Evaluate short-term post-surgical outcomes up to two months of hospital discharge  Other outcomes: please refer to Table 3 and Table 4 for further details. |
| Participant timeline | 13 | We expect that each study participant remains in the trial for approximately 60 days (10 days as an inpatient without any major complications and an expected two-month post-surgery for a follow-up visit).  (Please refer to Figure 1 and/or Figure 2 for further details). |
| Sample size | 14 | Total sample size: 30 study participants (12 patients, 12 significant others and 6 critical care nurses).  Justification: study sample size was based on recommended literature and on previous feasibility studies. In addition, this decision involved the consideration of the planned study intervention’s complexity, funding, study design and timelines. |
| Recruitment | 15 | The lead researcher will screen pre-operative cardiac clinic lists, cardiothoracic theatre lists and attend weekly multidisciplinary team meetings to identify potential suitable participants. |
| **Methods: Assignment of interventions (for controlled trials)** | | |
| Allocation: |  |  |
| Sequence generation | 16a | N/A |
| Allocation concealment mechanism | 16b | N/A - All study patients will receive the same intervention, without randomisation or blinding. |
| Implementation | 16c | N/A |
| Blinding (masking) | 17a | N/A |
|  | 17b | N/A |
| **Methods: Data collection, management, and analysis** | | |
| Data collection methods | 18a | Study data collection will include gathering data through qualitative interviews, quality of life questionnaires and patient’s medical records in line with the schedule of assessments.  (Please refer to Figure 1 and Table 4 for further information). |
|  | 18b | Those participants wishing to withdraw will be asked for permission to keep the data already collected, but no further research procedures will be carried out in relation to these patients.  Any protocol deviations will be documented in a protocol deviation form and safely filed in the Trial Master File. Corrective actions and preventative actions will be employed to avoid new deviations from the study protocol. |
| Data management | 19 | The research team will follow the principles of Good Clinical Practice (GCP), the EU General Data Protection Regulation (GDPR) and UK Data Protection Act 2018 to ensure all the data collected will be safely protected. All data collected will be kept on a Sponsor password protected NHS computer. Only members of the study delegation log will have access to these electronic files. Original consent forms will be kept in the investigator site file (ISF) and one copy will be uploaded in the patients' electronic notes. The ISF will be kept with the Chief Investigator, in a key-locked cabinet inside a key locked office, accessible only to members of the research team who have signed the delegation log.  Personal pictures/videos will be sent to an encrypted NHS email and stored in a Sponsor password protected NHS computer. Subsequently, pictures/videos will be securely uploaded in line with Sponsor’s information governance to the respective research equipment (e.g., iPad, digital photo frame). Interviews will be audio-recorded using an encrypted research study dictaphone and data anonymised before being stored in a Sponsor password protected NHS computer. |
| Statistical methods | 20a | Evaluating study’s feasibility and acceptability will involve performing both qualitative and quantitative analysis.  (Please refer to the Data Analysis section). |
|  | 20b | Due to the study's small sample size, both qualitative and quantitative findings will be analysed and reported with careful consideration of their limitations. The research team might conduct appropriate inferential statistical tests, such as Fisher’s exact test for categorical variables and Wilcoxon test for continuous variables. Additionally, correlations may be performed to examine the relationships between vital signs, blood tests, ICU complications, mortality rates and the development of ICU delirium post-cardiac surgery. |
|  | 20c | Any missing data will be detailed individually due to the study's small sample size. |
| **Methods: Monitoring** | | |
| Data monitoring | 21a | Study outcomes will be regularly monitored and reported within the research team:  - Principal Investigator: Maria Reguenga  - Chief Investigator: Sanooj Soni  - Co-Investigators: Professor Stephen Brett, Professor Natalie Pattison and Smaragda Lampridou.  (Please refer to 21b for reporting study outcomes to Sponsor and Funders) |
|  | 21b | An annual/end-of-study report will be sent to both the Sponsor and UK Research Ethics Committee (REC), as well as to ClinicalTrials.gov. The funders (IHC) also require an interim and study closure reports. |
| Harms | 22 | The Sponsor, Imperial College Healthcare NHS Trust, holds a standard NHS hospital indemnity and insurance cover with NHS Resolution for NHS Trusts in England, which apply to this study. However, considering the nature of the study design, we do not expect any adverse or serious adverse events. Any events of clinical interest will be discussed within the research team, escalated to the clinical team and documented on participants’ medical notes, if appropriate. |
| Auditing | 23 | The study may be subject to audit by the Sponsor and other regulatory bodies to ensure adherence to GCP and the UK Policy Framework for Health and Social Care Research. Direct access will be granted to authorised representatives from the Sponsor, partner institution (Imperial College of London) and the regulatory authorities to allow any trial related monitoring, audits and inspections. The Sponsor may also carry out audits to ensure compliance with the protocol and appropriate local regulations. |
| Ethics and dissemination | | |
| Research ethics approval | 24 | The study obtained favourable approval from the Bradford and Leeds REC and Health Research Authority (HRA) in January 2024, with reference number 24/YH/0011. The study also received NHS sponsorship and Research Information Governance approval in February 2024, together with a Confirmation of Capacity and Capability to conduct the study at ICHT (23HH8130). The study will be conducted in accordance with the recommendations for physicians involved in research on human subjects adopted by the 18th World Medical Assembly, Helsinki 1964 and later revisions. |
| Protocol amendments | 25 | Every care was taken when drafting the study protocol, but corrections or amendments may be necessary in the future. No amendments to this protocol will be made without prior consultation and agreement with the Sponsor. Any amendments to the study that appear necessary during the study must be discussed within the Research Team and the Sponsor concurrently. If an agreement is reached concerning the need for an amendment, a new ethical and regulatory approval will be submitted to REC/HRA. |
| Consent or assent | 26a | Consent will happen after a full explanation has been given, an information leaflet offered, and time allowed for consideration and questions. The lead researcher (principal investigator) will request a signed consent form before study enrolment. |
|  | 26b | In occasions where participants are unable to consent or have lost their capacity to consent more than once whilst in ICU (e.g., delirium), their participation in the study will be considered by approaching their personal consultee (e.g., relative, friend, partner). Additionally, a Patient Regaining Capacity consent form will be signed post-operatively and, ideally before patients resume the study in the ICU, or after any periods of lacking capacity (e.g., surgery). |
| Confidentiality | 27 | The CI and all members of the delegated study team will preserve the confidentiality of participants taking part in the study. All data will be collected and managed in line with the Data Protection Act 2018 and GDPR. The risk of confidentiality breach will be minimised by ensuring all study-related data is stored securely in a pseudonymised form and participants will be identified by a study ID number only. Any patient-identifiable data that is shared with parties outside of the research study team will be modified such that it is in a linked anonymised form (with removal of patient hospital number, NHS number, name, date of birth, etc.). Additionally, the research team will ensure that any patient-identifiable data is safely kept in a key-locked cabinet only accessible to members of the study team and remaining study documentation is always securely stored in a password protected computer and/or research office. |
| Declaration of interests | 28 | This study is being undertaken as part of a Pre-Doctoral Research Fellowship that has been awarded to the PI, Maria Reguenga, by Imperial Health Charity and is being funded by the NIHR Imperial Biomedical Research Centre.  We currently do not have funding to support the participation of patient, family members and healthcare professionals. Additionally, other members of the research team will not receive any money contributions from supporting this study. |
| Access to data | 29 | Only staff on the delegation log will have access to the study data. Participants’ folders will be retained in the key-protected research offices, within the Sponsor’s premises. |
| Ancillary and post-trial care | 30 | Following data analysis, dissemination and study closure, the research team will retain essential documents until notified by the Sponsor and then archived for at least ten years after study completion, as per Sponsor archiving policy. Study files and other source data (including copies of protocol, PIS, records of informed consent, and other documents pertaining to the conduct of the study) will be kept for the maximum period of time permitted by the institution. |
| Dissemination policy | 31a | Study results will be shared within a patient and public involvement-focused group involved in the co-design of the study intervention and with study participants upon written request. Study insights will also be communicated within local research-led meetings and our local cardiac ICU. |
|  | 31b | We intend to disseminate the study findings via peer-reviewed open-access journals and conduct oral presentations at both national and international research-led meetings within the critical care cardiac field. |
|  | 31c | N/A |
| Appendices |  |  |
| Informed consent materials | 32 | Please refer to:  S2 File. PIS for Patients  S3 File. PIS for Family Members or Friends  S4 File. Information Sheet for Personal Consultees  S5 File. PIS for Critical Care Nurses |
| Biological specimens | 33 | N/A |

*It is strongly recommended that this checklist be read in conjunction with the SPIRIT 2013 Explanation & Elaboration for important clarification on the items. Amendments to the protocol should be tracked and dated. The SPIRIT checklist is copyrighted by the SPIRIT Group under the Creative Commons “[Attribution-NonCommercial-NoDerivs 3.0 Unported](http://www.creativecommons.org/licenses/by-nc-nd/3.0/)” license.
