# Supplementary material for: The use of audio-visual aids to reduce delirium after cardiac surgery in intensive care units (DaCSi-ICU): A feasibility study protocol
Source: PLoS One. 2025 Apr 24;20(4):e0320935. doi: 10.1371/journal.pone.0320935 (PMC12021270; doi:10.1371/journal.pone.0320935)
Supplement: S4 File — (PDF) [file pone.0320935.s008.pdf]

PERSONAL CONSULTEE INFORMATION SHEET AND DECLARATION FORM

Study title

DaCsi-ICU: The use of auditory-visual stimulation to reduce delirium rates in intensive care patients post-cardiac surgery: a feasibility study

Introduction

We feel your relative/friend is unable to decide for himself/herself whether to participate in this research.

To help decide if he/she should join the study, we'd like to ask your opinion whether or not they would want to be involved. We'd ask you to consider what you know of their wishes and feelings, and to consider their interests. Please let us know of any advance decisions they may have made about participating in research. These should take precedence.

If you decide your relative/friend would have no objection to taking part we will ask you to read and sign the consultee declaration on the last page of this information leaflet. We'll then give you a copy to keep. We will keep you fully informed during the study so you can let us know if you have any concerns or you think your relative/friend should be withdrawn.

If you decide that your friend/relative would not wish to take part it will not affect the standard of care they receive in any way.

If you are unsure about taking the role of consultee you may seek independent advice.

We will understand if you do not want to take on this responsibility.

What is the purpose of the study?

After major cardiac surgery, it is not uncommon for patients to become confused and disoriented. This is called 'delirium'. More than half of patients undergoing heart surgery (up to 55%), experience confusion in the intensive care unit (ICU). This research will use a simple intervention to try to reduce the frequency, severity and duration of this condition.

The research team plans to explore and create an audio-visual (sound/sight) package involving family members/friends or patients to possibly prevent and manage delirium in ICU. This package will include pre-selected pictures and family videos, which will be used following the surgery.

We think that the combination of selected photos and family videos will promote your significant other's awareness of time, date and location (reorientation) after the long surgery. We also believe that this package will contribute to the sensation of comfort and confidence (reassurance) when your significant other faces an unfamiliar environment. If so, we hope that this will lead to a reduction of ICU delirium, fewer post-operative complications and better health outcomes after hospital discharge.

Why has your significant other been chosen?

Your relative/friend/partner has been chosen pre-operatively because they were identified as a patient, who underwent cardiac surgery and is at risk of developing ICU delirium.

About 12 patients in the UK are expected to participate in the study. The duration of their participation is expected to be up to two months.

Does your significant other have to take part?

No, your friend/relative/partner does not have to continue taking part in the study. We are asking your opinion as to whether you think your significant other would want to remain in the study.

If you think that they would like to continue in the study, you will be given this information sheet to keep and be asked to sign a declaration form. Your friend/relative/partner is still free to withdraw at any time and without giving a reason to do so. A decision to withdraw at any time, or a decision not to take part, will not affect the standard of care your friend/relative/partner will receive in the future.

What will happen to your significant other takes part?

Your friend/relative/partner has already been approached to take part in the study before their operation. As they have agreed to take part, they were attributed with a study ID number to keep their data anonymised (personally identifiable information is removed). They have also selected a family member or close friend to participate in this project as well.

During the pre-operative stage, your friend/relative/partner has selected approximately 10 pictures, including photos of themselves alone, with family members and/or friends. Their selected family member or friend has also self-recorded a total of 6-9 videos based on a guided script promoting time/date orientation and providing reassurance messages.

Before surgery, the research team safely uploaded the pictures and videos to the password-protected electronic devices (e.g., iPad), which was completed according to the Imperial College Healthcare Trust and the Research Governance Integrity Team guidelines.

With your friend/relative/partner consent, selected photos and family videos will be displayed at the bedside, once they are awake from the general anaesthetic after the surgery. The pictures will be continuously shown on a digital photo device during daytime (8am-8pm). Family videos will be played at least three times a day (at 9am, 2pm and 7pm) on an electronic device (tablet/iPad) during their stay in ICU. Upon their request, we will also show them the pictures and/or videos outside the planned routine schedule and overnight.

If you think they would like to continue receiving the study intervention when they suffer from periods of acute confusion (delirium) and are unable to decide for themselves, we will continue to show your friend/relative/partner the pictures and videos according to the planned schedule and during the delirium episodes.

As discussed with your friend/relative/partner before the surgery, during ICU we will also access medical notes to keep a record of their observations (e.g., heart rate, blood pressure, etc.) and other outcome data (e.g., blood test results, delirium status, etc.), which is collected by nurses daily. The study intervention (pictures/videos) will finish upon their ICU discharge.

The study team will also keep all documents and information collected safe and only accessible to members of the study team. We will be also responsible for deleting all family videos and personal photos from used devices upon your friend/relative/partner ICU discharge. The research team will not be entitled to keep any copies of videos and photos, without seeking their prior consent.

What is the drug or intervention that is being tested?

This study does not involve the administration or change of medications/drugs. This study aims to test the efficacy of an innovative programme (how well the intervention works) in preventing and managing delirium in ICU.

The intervention being tested involves the implementation of an audio and visual package in ICU, which includes a combination of selected pictures and family videos. Pictures will be played on a digital photo frame and videos on a digital tablet/iPad (small screen).

What are the alternatives for diagnosis or treatment?

Participation in the study is in addition to having surgery and not a replacement for surgery. A decision to not take part in this study, will not affect the standard of care that you or your significant other will receive in the future or your employment in any way.

What are the side effects of any treatment received when taking part?

Participation in the study may trigger feelings that your significant other finds it difficult to manage. But they might also find it helpful to discuss this feelings within a supportive environment and with the research team.

Due to the nature of the study design, the research team does not expect any other side effects from taking part in the study.

What are the possible disadvantages and risks of taking part?

The risks of taking part in this study are minimal. Your significant other may potentially find it difficult or upsetting when watching the family videos or pictures. If so, they will be given time to pause and discuss their experiences with the study team.

If you feel that their participation in this study puts them at risk, please contact the study team by referring to the contact information at the end of the document.

What are the possible benefits of taking part?

As this study does not involve any treatment (e.g., medication), there is no direct benefit for them to continue taking part. We cannot promise the study intervention will help them, but the information we will get might help improve the pathway of care for future patients diagnosed with ICU delirium after cardiac surgery. They might also find it helpful to talk about their experiences with the research team.

Sometimes during the course of a research project, new information becomes available about the intervention that is being studied. If this happens, the research team will inform you about it and discuss with you whether you think they should remain or not in the study. If you decide that they should be withdraw from the study, the research team will make arrangements for their standard of care to continue.

What happens when the research study stops?

We will destroy sensitive information (e.g., contact details) shortly after your friend/relative/partner completes all the study tasks.

The research team will also be responsible for archiving information collected throughout the study at Imperial College Healthcare NHS Trust. All information will be kept inside a key and/or password-locked office and stored for ten years following the end of the study.

What happens when your friend/relative/partner regains consent?

We will ask your friend/relative/partner permission to use the data that we have collected during their ICU stay. They are able to refuse at any point and we will remove them and their data from the study. If they agree to continue in the study, they will be asked to sign a consent form.

What if something goes wrong?

Imperial College Healthcare NHS Trust holds standard NHS Hospital Indemnity and insurance cover with NHS resolution for NHS Trusts in England, which applies to this study. This does not affect your legal rights to seek compensation.

If your friend/relative/partner is harmed due to someone’s negligence, then they may have grounds for legal action. Regardless of this, if you wish to complain, or have any concerns about any aspect of the way you or your friend/relative/partner have been treated during the course of this study then you should immediately inform the Principal Investigator (contact details are outlined at the end of this information sheet).

The normal National Health Service complaints mechanisms are also available to you. If you are still not satisfied with the response, you may contact the Imperial AHSC Research Governance and Integrity Team.

How will we use information about your friend/relative/partner?

We will need to use information from your friend/relative/partner for this research project. This information will include your:

- Hospital number
- NHS number
- Contact details (e.g., telephone number, email address and home address)
- Medical notes

People will use this information to do the research or to check your friend/relative/partner records. People who do not need to know who your friend/relative/partner is will not be able to see their name or contact details. Your friend/relative/partner information will have a code number instead.

We will keep all information about your friend/relative/partner safe and secure. Once we have finished the study, we will keep some information so we can check the results. We will write our reports in a way that no one can work out that your friend/relative/partner took part in the study.

What are your choices about how your friend/relative/partner information is used?

Your friend/relative/partner can stop being part of the study at any time, without giving a reason, but we will keep information about them that we already have. Should them or you chose to withdraw them from the study, the data already collected about your friend/relative/partner will be maintained for study purposes, but no further study activities will

be conducted beyond that point. We need to manage their records in specific ways for the research to be reliable. This means that we will not be able to let you see or change the data we hold about your friend/relative/partner.

For any data protection enquires related with this research study, feel free to email study team or alternatively, the ICHT Data Protection Office at imperial.dpo@nhs.net.

Where can you find out more about how your friend/relative/partner information is used?

You can find out more about how we use your information:

- at www.hra.nhs.uk/information-about-patients/
- by asking one of the research team
- by sending an email to maria.reguenga@nhs.net, or
- by ringing us on 020 331 31703.

What will happen to the results of the research study?

Once the study has finished, we will be happy to make the results available to your friend/relative/partner. We aim to disseminate the results to other healthcare professionals via publication of the results in journals and presentations at various conferences. In this context, direct quotes sourced from interviews will be utilised and subsequently published in peer-reviewed journals.

If your friend/relative/partner opt to remain on the contact list until the study results are published and shared with participants, you will be asked to sign an extra optional clause in the consent form below and your contact details will be then destroyed upon study closure.

Who is organising and funding this study?

This research is supported by the Imperial Health Charity Pre-Doctoral Research Fellowship funded by the NIHR Imperial Biomedical Research Centre. It is being conducted by a Research Fellow within the Critical Care Department at Imperial Healthcare NHS Trust. Participants will not be paid for taking part in the study.

Who has reviewed the study?

This study was given a favourable ethical opinion for conduction within the NHS by the Research Ethical Committee (REC) of Bradford-Leeds.

Contact for further information

The researchers involved in this study are the following people:

- Professor Stephen Brett, Professor of Critical Care & Consultant at the Intensive Care Unit in Hammersmith Hospital, Imperial College Healthcare NHS Trust.
- Dr. Sanooj Soni, Consultant at the Intensive Care Unit in Hammersmith Hospital, Imperial College Healthcare NHS Trust.
- Professor Natalie Pattison, Research Fellow in Residence with a joint appointment between Imperial College of London and Imperial College Healthcare NHS Trust.
- Smaragda Lamperidou, Research Fellow at the Vascular Surgery Department at Imperial College of London.

- Maria Reguenga, Research Fellow in Critical Care at the Intensive Care Unit in Hammersmith Hospital, Imperial College Healthcare NHS Trust.

If you require any further information the Principal Investigator, Maria Reguenga, can be contacted by email at maria.reguenga@nhs.net or by phone on 020 331 31703.

If you wish to complain, the normal National Health Service complaints mechanisms are available to you. If you are not satisfied with the response of the above, you can also contact the Joint Research Compliance office on Tel: 0207 594 9459/ 0207 594 1862. Additionally, you can also contact the Imperial College Healthcare NHS Trust’s Patient Advice and Liaison Service (PALS) (E-mail: PALS@imperial.nhs.uk).

CONSULTEE DECLARATION FORM

Full Title of Project: DaCsi-ICU: The use of auditory-visual stimulation to reduce delirium rates in intensive care patients post-cardiac surgery: a feasibility study

Principal Investigator: Maria Reguenga

Participant ID: ____________

Please initial box

1. I _________________________________ [name of consultee] have been consulted about _______________________________ [name of potential participant]'s participation in this research project. I have had the opportunity to ask questions about the study and understand what is involved. ☐
2. In my opinion he/she would have no objection to taking part in the above study. ☐
3. I understand that I can request for he/she to be withdrawn at any time, without giving any reason and without his/her medical care or legal rights being affected. ☐
4. I understand that relevant sections of his/her care record and data collected during the study may be looked at by responsible individuals from Imperial College Healthcare NHS Trust or from regulatory authorities, where it is relevant to their taking part in this research. ☐
5. I agree to their GP or other care professional being informed of their participation in the study. ☐
6. I understand that the information collected about him/her will be used to support other research in the future, and may be shared anonymously with other researchers. ☐

________________________	________________	________________
Name of personal consultee	Signature	Date
________________________	________________	________________
Name of person taking consent<br>(if different from Principal Investigator)	Signature	Date
