# Supplementary material for: The use of audio-visual aids to reduce delirium after cardiac surgery in intensive care units (DaCSi-ICU): A feasibility study protocol
Source: PLoS One. 2025 Apr 24;20(4):e0320935. doi: 10.1371/journal.pone.0320935 (PMC12021270; doi:10.1371/journal.pone.0320935)
Supplement: S5 File — (PDF) [file pone.0320935.s009.pdf]

PARTICIPANT INFORMATION SHEET AND INFORMED CONSENT FORM

Study title

DaCsi-ICU: The use of auditory-visual stimulation to reduce delirium rates in intensive care patients post-cardiac surgery: a feasibility study

Invitation paragraph

You are being invited to take part in a research study. Before you decide, it is important for you to understand why the research is being done and what it will involve. Please take the time to read the following information carefully and discuss it with others if you wish. Ask a member of the research team if there is anything that is not clear or if you would like more information. Take as much time as you need to decide whether or not you wish to take part.

If you agree to take part, you will be asked to fill out, sign and date this information sheet and consent form and to keep it as a useful reference on the study details and research contacts.

Thank you for reading this.

What is the purpose of the study?

After major cardiac surgery, it is not uncommon for patients to become confused and disoriented. This is called 'delirium'. More than half of patients undergoing heart surgery (up to 55%), experience confusion in the intensive care unit (ICU). This research will use a simple intervention to try to reduce the frequency, severity and duration of this condition.

Family involvement is considered to be one of the most effective interventions in reducing the incidence of delirium in ICU. To explore this, we will investigate whether a family-focused audio-visual sensory stimulation package could potentially be used in the ICU to effectively prevent and manage delirium. This package will include selected pictures and family videos, which will be played to patients following their elective major cardiac surgery (e.g., CABG).

We hypothesise that personal photos and family videos will help to promote reorientation and reassurance in patients when facing an unfamiliar environment. If so, we hope that this will lead to a reduction of ICU delirium, fewer post-operative complications and better health outcomes after hospital discharge.

Why have I been chosen?

You have been invited because you were identified as one of the critical care nurses, who have provided directed care to at least one of the patients who received the study intervention.

We aim to recruit 6 healthcare professionals to participate in the study. The duration of your participation is expected to be up to a week.

Do I have to take part?

No, it is up to you to decide whether or not to take part. If you do decide to take part, you will be given this information sheet to keep and be asked to sign a consent form. If you decide to

take part, you are still free to withdraw at any time and without giving a reason. A decision to withdraw at any time, or a decision not to take part, will not affect your employment status or the standard of care you will receive in the future.

What will happen to me if I take part?

You will be approached to take part in the study after you have provided directed care to at least one of the patients who received the study intervention in the ICU. You will be given at least 24 hours to consider your participation in the study. If you agree to take part in the study, you will be attributed with a study ID number so your data is anonymised (personally identifiable information is removed).

Secondly, you will be asked to complete one interview with a member of the research team up to a week after you have been enrolled on the study. We aim to conduct the interview face-to-face, which will happen in a private room in the ICU at Hammersmith Hospital. However, the option to interview you remotely (e.g., from home using a phone or computer) will be given considering your preference.

During the face-to-face interview, the research team will follow social distancing guidance and appropriate personal protective equipment where necessary. On the day of your interview, you will be asked to confirm whether you are still happy to participate in the study. Throughout the interview, we will discuss your experience in delivering the intervention and/or patient experience whilst they stay in the ICU.

The interview will run for approximately 20 minutes and, we will aim to cover three main topics:

- The contributions and barriers of this package being routinely delivered in ICU;
- The impact of the family video and photos on patients' orientation and reassurance;
- Your overall experience as a nurse delivering the intervention in ICU.

To help with the analysis of the results, the research team will audio-record interviews and recordings will be then pseudonymised. Recordings from the interview will be stored on an Imperial College Healthcare NHS Trust (ICHT) computer that is password-protected. We will keep the recordings until they have been transcribed and once this is complete, we will destroy them appropriately.

What will I have to do?

You must be willing to comply with the study tasks (e.g., interview) and to help the research team with the information they need to know about you. All information will be kept confidential and not be shared outside the research team without your prior consent.

You will continue to receive standard of care from your GP and NHS hospitals. Taking part in this study will not affect any of your usual everyday activities or your employment; and you should also continue to take your medications as directed

What is the drug or intervention that is being tested?

This study does not involve the administration or change of medications/drugs. This study aims to test the efficacy of an innovative programme (how well the intervention works) in preventing and managing delirium in ICU.

The intervention being tested involves the implementation of an audio and visual package in ICU, which includes a combination of selected pictures and family videos. Pictures will be played on a digital photo frame and videos on a digital tablet/iPad (small screen).

What are the alternatives for diagnosis or treatment?

Participation in the study is in addition to having surgery and not a replacement for surgery. A decision to not take part in this study, will not affect the standard of care that you will receive in the future or your employment in any way.

What are the side effects of any treatment received when taking part?

Participation in the study may trigger feelings that critical care nurses find difficult to manage. But you might also find it helpful to discuss this within a supportive environment and with the research team.

Due to the nature of the study design, the research team does not expect any other side effects in taking part in the study.

What are the possible disadvantages and risks of taking part?

The risks of taking part in this study are minimal. The study requires a commitment to complete one single interview with a member of the research team. Thus, the only disadvantage would be the time spent in completing the study tasks. You may potentially find it difficult or upsetting when discussing your experiences, if so, you are free to pause or stop at any point in an interview. There will also be time allocated at the end of interviews to ask any further questions or advice.

If you feel that participation in this study puts you at risk, please contact the study team by referring to the contact information at the end of the document.

What are the possible benefits of taking part?

As this study does not involve any treatment (e.g., medication), there is no direct benefit to you in taking part. We cannot promise the study will help you but the information we will get might help improve the pathway of care for future patients diagnosed with ICU delirium after cardiac surgery. You may also find it helpful to talk about your experiences with the research team during or after interviews.

Sometimes during the course of a research project, new information becomes available about the intervention that is being studied. If this happens, your research team will inform you about it and discuss with you whether you want to continue in the study. If you decide to withdraw the research team will make arrangements for your care to continue. If you decide to continue in the study, you will be asked to sign an updated consent form.

What happens when the research study stops?

We will destroy sensitive information (e.g., contact details) shortly after you complete all the study tasks.

The research team will also be responsible for archiving information collected throughout the study at Imperial College Healthcare NHS Trust. All information will be kept inside a key and/or password-locked office and stored for ten years following the end of the study.

What if something goes wrong?

Imperial College Healthcare NHS Trust holds standard NHS Hospital Indemnity and insurance cover with NHS resolution for NHS Trusts in England, which applies to this study. This does not affect your legal rights to seek compensation.

If you are harmed due to someone's negligence, then you may have grounds for legal action. Regardless of this, if you wish to complain, or have any concerns about any aspect of the way you have been treated during the course of this study then you should immediately inform the Principal Investigator (contact details are outlined at the end of this information sheet).

The normal National Health Service complaints mechanisms are also available to you. If you are still not satisfied with the response, you may contact the Imperial AHSC Research Governance and Integrity Team.

How will we use information about you?

We will need to use information from you for this research project. This information will include your:

- Name
- Contact details (e.g., telephone number, email address and home address).

People will use this information to do the research or to check your records. People who do not need to know who you are will not be able to see your name or contact details. Your information will have a code number instead.

We will keep all information about you safe and secure. Once we have finished the study, we will keep some information so we can check the results. We will write our reports in a way that no one can work out that you took part in the study.

What are your choices about how your information is used?

You can stop being part of the study at any time, without giving a reason, but we will keep information about you that we already have. Should you chose to withdraw from the study, the data already collected will be maintained for study purposes, but no further study activities will be conducted beyond that point. We need to manage your records in specific ways for the research to be reliable. This means that we will not be able to let you see or change the data we hold about you.

For any data protection enquires related with this research study, feel free to email study team or alternatively, the ICHT Data Protection Office at imperial.dpo@nhs.net.

Where can you find out more about how your information is used?

You can find out more about how we use your information:

- at www.hra.nhs.uk/information-about-patients/
- by asking one of the research team
- by sending an email to maria.reguenga@nhs.net, or
- by ringing us on 020 331 31703.

What will happen to the results of the research study?

Once the study has finished, we will be happy to make the results available to you. We aim to disseminate the results to other healthcare professionals via publication of the results in journals and presentations at various conferences. In this context, direct quotes sourced from interviews will be utilised and subsequently published in peer-reviewed journals.

If you opt to remain on the contact list until the study results are published and shared with participants, you will be asked to sign an extra optional clause in the consent form below and your contact details will be then destroyed upon study closure.

Who is organising and funding this study?

This research is supported by the Imperial Health Charity Pre-Doctoral Research Fellowship funded by the NIHR Imperial Biomedical Research Centre. It is being conducted by a Research Fellow within the Critical Care Department at Imperial Healthcare NHS Trust. Participants will not be paid for taking part in the study.

Who has reviewed the study?

This study was given a favourable ethical opinion for conduction within the NHS by the Research Ethical Committee (REC) of Bradford-Leeds.

Contact for further information

The researchers involved in this study are the following people:

- Professor Stephen Brett, Professor of Critical Care & Consultant at the Intensive Care Unit in Hammersmith Hospital, Imperial College Healthcare NHS Trust.
- Dr. Sanooj Soni, Consultant at the Intensive Care Unit in Hammersmith Hospital, Imperial College Healthcare NHS Trust.
- Professor Natalie Pattison, Research Fellow in Residence with a joint appointment between Imperial College of London and Imperial College Healthcare NHS Trust.
- Smaragda Lamperidou, Research Fellow at the Vascular Surgery Department at Imperial College of London.
- Maria Reguenga, Research Fellow in Critical Care at the Intensive Care Unit in Hammersmith Hospital, Imperial College Healthcare NHS Trust.

If you require any further information the Principal Investigator, Maria Reguenga, can be contacted by email at maria.reguenga@nhs.net or by phone on 020 331 31703.

If you wish to complain, the normal National Health Service complaints mechanisms are available to you. If you are not satisfied with the response of the above, you can also contact the Joint Research Compliance office on Tel: 0207 594 9459/ 0207 594 1862. Additionally, you can also contact the Imperial College Healthcare NHS Trust's Patient Advice and Liaison Service (PALS) (E-mail: PALS@imperial.nhs.uk).

Thank you for taking part in this study!

STUDY CONSENT FORM

Full Title of Project: DaCsi-ICU: The use of auditory-visual stimulation to reduce delirium rates in intensive care patients post-cardiac surgery: a feasibility study

Principal Investigator: Maria Reguenga

Please initial box

1. I confirm that I have read and understand the participant information sheet version .... dated .... for the above study and have had the opportunity to ask questions which have been answered fully. ☐
2. I understand that my participation is voluntary, and I am free to withdraw at any time, without giving any reason and without my medical care or legal rights being affected. ☐
3. I agree to comply with the different study tasks as the Patient Information Sheet suggests (e.g., interview). ☐
4. I understand that sections of any of my medical notes may be looked at by responsible individuals from Imperial College Healthcare NHS Trust or from regulatory authorities where it is relevant to my taking part in this research. ☐
5. I agree to the recording of the interview. Audio recordings will be kept until they have been transcribed; once this is complete, they will be destroyed. ☐
6. **OPTIONAL** - I agree / do not agree (delete as applicable) to being sent the study results once the study has finished. Contact details will be kept until the results are shared by the research team; once this is complete, they will be destroyed. ☐
7. I give / do not give (delete as applicable) consent to being contacted about the possibility to take part in other research studies. ☐
8. I consent to take part in the above study. ☐

___________________________	___________________	___________________
Name of participant	Signature	Date
___________________________	___________________	___________________
Name of person taking consent<br>(if different from Principal Investigator)	Signature	Date
