# Supplementary material for: The use of audio-visual aids to reduce delirium after cardiac surgery in intensive care units (DaCSi-ICU): A feasibility study protocol
Source: PLoS One. 2025 Apr 24;20(4):e0320935. doi: 10.1371/journal.pone.0320935 (PMC12021270; doi:10.1371/journal.pone.0320935)
Supplement: S6 File — (DOCX) [file pone.0320935.s010.docx]

**S6 File. Video Script for Family Members/Friends**

VIDEO GUIDED SCRIPT TEMPLATE FOR FAMILY MEMBERS/FRIENDS

**A) Reorientation**

- Start the video by greeting the patient and mentioning their name.

- Introduce yourself (e.g., name/preferred name) and your relationship to the patient.

- State where you are (e.g., house, garden, etc.) and if you are alone or accompanied (e.g., with significant others, pets, special objects/toys)

- Mention the patient’s name in the beginning/end of the next sentences

- Provide a reference to the approximate time (9am, 2pm or 7pm) and date (e.g., Wednesday)

- Mention that patients have now had the surgery and are staying in Intensive Care at Hammersmith Hospital in London.

- State how many days have been since surgery (e.g., today is day 1 after your cardiac surgery)

**B) Reassurance**

- Continue the video by explaining that this is a recorded video/message to help patients

understand what is going on around them.

- Family members to choose from the following options and/or free to personalise the next one to two final sentences of the video.

Examples of reassurance and supportive messages:

1. It is OK, do not be scared.
2. You will soon be home with us.
3. Your nurses and doctors are here looking after you.
4. You might be uncomfortable, but the nurses are giving you painkillers.
5. It is loud and noisy because of the machines that are helping you get better.
6. You may have some wires and tubes in place to help you recover.
7. You may have something on your wrists to keep you from pulling at the wires and tubes by accident, but will soon be removed.
8. You can’t talk right now because of your breathing tube, but the nurses know you might be uncomfortable and are giving you medicine for that.

- Finish the video with a farewell message (e.g. see you soon) and mention the patient's name at the beginning/end of the sentence.

**Thank you for taking part in this study!**
