# Supplementary material for: The use of audio-visual aids to reduce delirium after cardiac surgery in intensive care units (DaCSi-ICU): A feasibility study protocol
Source: PLoS One. 2025 Apr 24;20(4):e0320935. doi: 10.1371/journal.pone.0320935 (PMC12021270; doi:10.1371/journal.pone.0320935)
Supplement: S7 File — (DOCX) [file pone.0320935.s011.docx]

**S7 File. Study Daily Checklist**

| ***N*** | Daytime Checklist (8am – 8pm) | **Tick as appropriate** |
| --- | --- | --- |
| *1* | Did the patient have any unknown visual/auditory impairment? |  Yes  No |
| *2* | Were any visual/auditory aids (eg. glasses/hearing aids) in place during the intervention delivery? |  Yes  No  N/A |
| *3* | Was the family intervention delivered 3 times during the day (8am-8pm)? |  Yes  No  Times: 1. ____:____; 2.____:____;  3. ____:____ |
| *4* | Did the patient express any verbal/non-verbal emotions when watching the video? |  Yes  No  If “Yes”, please specify: ____ |
| ***5*** | Did the patient look at the digital photo frame during the day? |  Yes  No  If “Yes”, how many times: ____ |
| ***6*** | Did the intervention impact ICU nursing care? |  Yes  No  If “Yes”, please specify: ____ |
| ***7*** | Was the family intervention delivered without any untoward events (eg., acute distress)? |  Yes  No  If “No”, please specify: ____ |
| ***8*** | Did the patient request to watch the video more than 3 times a day? |  Yes  No  If “Yes”, please specify the total number of times: ____ |
| *9* | Did the patient develop delirium during the day (8am-8pm)? |  Yes  No  9a. If “Yes”, was the video played again?  Yes  No  9b. If “Yes”, did it improve delirium status?  Yes  No |

| ***N*** | Night-time Checklist (8pm – 8am) | **Tick as appropriate** |
| --- | --- | --- |
| *1* | Were any visual/auditory aids removed prior to sleep? |  Yes  No |
| *2* | From a nurse’s perspective, how many hours did the patient sleep? | ______ hours |
| ***3*** | Did the patient request to watch the video overnight? |  Yes  No  If “Yes”, how many times: ______ |
| *4* | Did the patient develop delirium overnight? |  Yes  No  4a. If “Yes”, was the video played again?  Yes  No  4b. If “Yes”, did it improve delirium status?  Yes  No |
| ***5*** | Was the iPad plugged in to charge overnight? |  Yes  No |
