# Supplementary material for: The use of audio-visual aids to reduce delirium after cardiac surgery in intensive care units (DaCSi-ICU): A feasibility study protocol
Source: PLoS One. 2025 Apr 24;20(4):e0320935. doi: 10.1371/journal.pone.0320935 (PMC12021270; doi:10.1371/journal.pone.0320935)
Supplement: S8 File — (PDF) [file pone.0320935.s012.pdf]

Imperial College London

Research Governance and Integrity Team

Imperial College Healthcare NHS Trust

DaCsi - ICU

Delirium after Cardiac surgery in Intensive Care Units

The use of auditory-visual stimulation to reduce delirium rates in intensive care patients post-cardiac surgery: a feasibility study

V1.3 01DEC2023

MAIN SPONSOR: Imperial College Healthcare NHS Trust

FUNDERS: Imperial College Charity

STUDY COORDINATION CENTRE: Imperial College Healthcare NHS Trust & Imperial College London

IRAS Project ID: 331314
REC reference: 24/YH/0011

Protocol authorised by:

Name & Role	Date	Signature

STUDY MANAGEMENT GROUP

Chief Investigator: Dr Sanooj Soni

Principal Investigator: Maria Reguenga

Co-investigators/Sub-Investigators: Professor Steve Brett and Professor Natalie Pattison

Study Management: Maria Reguenga

Study Coordination Centre: Hammersmith Hospital

For general/clinical queries, supply of study documentation, and collection of data, please contact:

Study Coordinator: Maria Reguenga

Tel: 0203 3131703

E-mail: maria.reguenga@nhs.net

Address: Imperial Centre for Translational and Experimental Medicine (ICTEM), Hammersmith Hospital (L Block), 72 Du Cane Rd, London, W12 0NN

Sponsor

Imperial College Healthcare NHS Trust is the main research Sponsor for this study. For further information regarding the sponsorship conditions, please contact the Head of Regulatory Compliance at:

Research Governance and Integrity Team
Imperial College London and Imperial College Healthcare NHS Trust
Room 215, Level 2, Medical School Building
Norfolk Place
London, W2 1PG
Tel: **0207 594 1862**
Imperial College - Research Governance and Integrity Team (RGIT) Website

Funder

This research study is being undertaken as part of a Pre-Doctoral Research Fellowship that has been awarded to the Principal Investigator (PI), Maria Reguenga, by the Imperial Health Charity (IHC) and is being funded by the National Institute for Health and Care Research (NIHR) Imperial Biomedical Research Centre (BRC).

The study has been peer-reviewed by the IHC fellowship committee before the decision to fund and the proposal has also been supported by the Intensive Care Lead Nurse at Hammersmith Hospital and members of the public with lived ICU experience.

Document Summary

This protocol describes the study of delirium in critical care patients' post-cardiac surgery and provides information regarding procedures necessary to enrol participants in this study. Every care was taken in its drafting, but corrections or amendments may be necessary. These will be circulated to investigators in the study prior to re-submission. Problems relating to this study should be referred, in the first instance, to the Chief Investigator (CI) and sub-investigators (SIs).

This study will adhere to the principles outlined in the UK Policy Framework for Health and Social Care Research. It will be conducted in compliance with the outline below protocol and other local Imperial College Healthcare NHS Trust (ICHT) regulatory requirements as appropriate. Data will be collected and managed according to the Data Protection Act 2018 and in line with the General Data Protection Regulation (GDPR).

TABLE OF CONTENTS

Pag.

1. INTRODUCTION 5
1.1. BACKGROUND 5
1.2. RATIONALE FOR CURRENT STUDY 5
2. STUDY OBJECTIVES 6
2.1. STUDY PRIMARY OBJECTIVES 6
2.2. STUDY SECONDARY OBJECTIVES 6
3. STUDY DESIGN 6
3.1. STUDY OUTCOME MEASURES 7
4. PARTICIPANT ENTRY 8
4.1. PRE-REGISTRATION EVALUATIONS 8
4.2. INCLUSION CRITERIA 8
4.3. EXCLUSION CRITERIA 8
4.4. WITHDRAWAL CRITERIA 9
5. ADVERSE EVENTS 13
6. STUDY ASSESSMENTS 13
7. STATISTICS AND DATA ANALYSIS 15
8. REGULATORY ISSUES 15
8.1. ETHICS APPROVAL 16
8.2. CONSENT 16
8.3. CONFIDENTIALITY 17
8.5. INDEMNITY 19
8.6. SPONSOR 19
8.7. FUNDING 19
8.8. AUDITS 19
9. STUDY MANAGEMENT 19
10. PUBLICATION POLICY 20
11. REFERENCES 20

GLOSSARY OF ABBREVIATIONS

BRC	Biomedical Research Centre
CAM-ICU	Confusion Assessment Method
CI	Chief Investigator
GCP	Good Clinical Practice
GDPR	General Data Protection Regulation
GP	General Practitioners
IHC	Imperial Health Charity
NHS	National Healthcare Service
HRA	Health Research Authority
ICHT	Imperial College Healthcare NHS Trust
ICF	Informed Consent Form
ICU	Intensive Care Unit
ISF	Investigator Site File
QoL	Quality of Life
NIHR	National Institute for Health and Care Research
PI	Principal Investigator
PIS	Patient Information Sheet
PPI	Patient and Public Involvement
RASS	Richmond Agitation-Sedation Scale
REC	Research Ethics Committee
SIs	Sub-Investigators
SoA	Schedule of Assessments

KEYWORDS

Critical care; Delirium; Family involvement; Non-pharmacological interventions; Nurses

STUDY SUMMARY

**TITLE**	DaCSi-ICU: The use of auditory-visual stimulation to reduce delirium rates in intensive care patients' post-cardiac surgery: a feasibility study
**DESIGN**	This study is a mixed methods research design that includes collection of data through interviews, quality of life questionnaires and patients' medical records.
**AIMS**	▪ Determine the incidence of ICU delirium in ICHT following cardiac surgery<br>▪ Explore the compliance of outcome measures that diagnose ICU delirium<br>▪ Implement a family-focused sensory stimulation programme in the ICU<br>▪ Evaluate its useability and potential impact in patients, families and ICU staff
**OUTCOME MEASURES**	This study endpoints are to:<br>1. Assess the useability/practicalities of implementing a family-focused sensory stimulation to improve ICU delirium outcomes in patients undergoing cardiac surgery<br>2. Develop an innovative nurse-led tool to improve delirium outcomes in ICU post-cardiac surgery<br>3. Explore the acceptability of a new programme to be implemented in cardiac ICU, amongst critical care staff, patients and family members/friends
**POPULATION**	30 study participants
**ELIGIBILITY**	Study eligibility criteria is specific for each care group.
**DURATION**	24 months at Hammersmith Hospital, ICHT

1. INTRODUCTION

Delirium can affect over 50% of patients following cardiac surgery in intensive care units (ICU), leading to long-term cognitive impairment, prolonged hospital stays and increased costs to the NHS. Nurse-led auditory-visual stimulation to help prevent and manage ICU delirium is a relatively novel and unexplored clinical practice in the postoperative cardiac field.

This study aims to assess the feasibility of an innovative intervention using family focused sensory stimulation to reduce delirium in ICU patients following major cardiac surgery and improve post-surgical outcomes.

1.1. BACKGROUND

Delirium is a common and often undiagnosed brain dysfunction that occurs in ICU and is associated with increased morbidity and mortality [1,2]. Its incidence can vary between 30-80% depending on various risk factors, doubling the National Health Service (NHS) inpatient cost per patient compared to those without delirium [3,4]. It is a complex, distressing and debilitating condition that impacts patients, family members and healthcare professional [5].

Patients undergoing cardiac surgery are a particularly vulnerable group as ICU delirium affects up to 55% of these patients, due to operative reasons including surgical complexity and cardiac bypass time [6,7]. Delirium is associated with increased anxiety and depression, poor functional status, increased risk of stroke and significant cognitive impairment up to a year post-cardiac surgery. It has also been linked with an elevated number of hospital readmissions, longer ICU and hospital lengths of stay [8,9].

Given the significant burden on patients and the NHS, delirium prevention and management is considered to be national priority in ICU by the James Lind Alliance [10]. Delirium is potentially preventable and healthcare professionals must diagnose this condition in critical care units by using the Confusion Assessment Method (CAM-ICU) screening tool as per the UK National Institute for Health and Care Excellence (NICE) guidelines [11,12]. Whilst pharmacological interventions are the mainstay of treatment in ICU, the Society of Critical Care Medicine highlights the importance of non-pharmacological therapies in the management of ICU delirium [2]. Therefore, there is an urgent unmet need to identify novel non-pharmacological management strategies to prevent and treat ICU delirium [10].

1.2. RATIONALE FOR CURRENT STUDY

In recent years, family involvement has become a major theme in the literature when debating about patient outcomes in the healthcare system. Several studies developed in intensive care units have demonstrated positive results when involving family members in patient’s care plan. More analysis has shown the possible combination between family involvement and sensory stimulation when reducing anxiety and depression in ICU patients [8].

An intriguing potential therapy is the use of auditory and visual sensory stimulation combined with family involvement/interventions, which could potentially improve delirium outcomes. A meta-analysis demonstrated that non-pharmacologic therapies involving family members leads to a 24% risk reduction of ICU delirium [13]. A similar analysis shown that family involvement

in patient care was the most effective intervention in reducing ICU delirium incidence, compared to sedation reduction and exercise programmes [14]. Another study also concluded the reduction of delirium in ICU patients when exploring the impact of an auditory reorientation intervention involving family members [15]. Additionally, a feasibility study reported the acceptance by elderly patients and hospital staff of a family auditory and visual sensory intervention that was implemented in ward-based patients with dementia [16].

There is a paucity of information on this type of interventions, especially in ICU patients post cardiac surgery. Therefore, further studies are urgently required to investigate the use of family-centred sensory stimulation in ICU delirium prevention and management following major cardiac surgery.

2. STUDY OBJECTIVES

Research question: Is a family focussed auditory-visual stimulation feasible and acceptable to be delivered in a critical care setting to patient's post-cardiac surgery to reduce ICU delirium?

2.1. STUDY PRIMARY OBJECTIVES

Based on the research question, the primary aim of this research study is to:

- Evaluate the feasibility and acceptability of introducing an auditory and visual sensory stimulation intervention involving family members as part of prevention and management strategies for ICU delirium in patients following cardiac surgery.

2.2. STUDY SECONDARY OBJECTIVES

The secondary objectives to this study are to:

- Determine the incidence of newly diagnosed ICU delirium patients following cardiac surgery in the Imperial College Healthcare NHS Trust (ICHT);
- Investigate the compliance of primary outcome measures that diagnose patients with ICU delirium;
- Implement a sensory stimulation programme with family-focused auditory-visual cues in ICU;
- Assess the practicalities of implementing a sensory simulation programme and the potential impact on patients, family members and critical care staff.

3. STUDY DESIGN

The study will be conducted over twelve months in the Cardiac Intensive Care Unit at Hammersmith Hospital, Imperial College Healthcare NHS Trust. For this study, a total of 30 individuals (12 patients, 12 family members or close friends and 6 critical care nurses) will be recruited and interviewed to allow sufficient depth and richness of data. Patients and family members/friends will be mainly recruited from the ICHT pre-operative clinic outpatient department, which runs several pre-operative clinics per week. Each week, approximately eight patients undergo cardiac surgery, and the research team intends to recruit one patient and their family member/friend per a week for a period of 4 months.

All participants will be given time to consider their participation in the study and if happy will be consented by PI or other delegated member of the research team. Patients will be approached during one of their pre-operative clinic appointments and informed consent will be sought at this stage. Once written consent has been obtained, patients will be asked to nominate a family member or friend to be contacted by the PI. After signing the consent form, family members/friends will also be enrolled into the study and requested to self-record short videos pre-operatively (based on a guided script - ANNEX XIII), which will be safely uploaded to an iPad. In addition, patients will be requested to select multiple pictures, which will be securely uploaded into a digital photo frame. This package combination of videos and pictures will be shown to patients post-operatively in ICU. Critical care nurses will be recruited after they have provided direct care to at least one participant, who received the study intervention in ICU.

This is a mixed methods research study including the collection of data from patient's medical notes, interviews and quality of life questionnaires in accordance with the schedule of assessments (SoA, Annex I). This study also involves the implementation of an auditory-visual sensory stimulation package in the cardiac ICU at ICHT to participants enrolled in the study. Digital photos will be shown continuously at the patient bedside after cardiac surgery and will always have reference to time and date. Additionally, videos will be played throughout participants' stay in ICU for at least three times per day and upon participant request. Videos will be also played when participant show signs of ICU delirium with the aim of reducing delirium incidence and improve overall better health outcomes. Simultaneously, primary outcomes measures (CAM-ICU, a routine delirium screening measure) will be collected by the research team, as this is evaluated twice a day as per usual standard of ICU practice, with the aim of assessing whether patients develop delirium during their stay in ICU.

Interviews with patients and families/friends will be held in three different occasions. The first one will be on the same day as ICU discharge, the second interview will be on the day of hospital discharge and the third one will be carried out up to a month following hospital discharge during their clinical follow-up appointment. Where possible, family members/friend and patient's interviews will be conducted together (dyadic interviewing), but the possibility of performing interviews separately will be given, so that participants feel able to discuss opinions freely without influence. Nurses' interviews will be held as a one-off and after they have provided direct care to participants who received the proposed intervention.

Each patient will be followed up daily on the wards and once at the monthly follow-up clinic up after ICU discharge, where cognitive and emotional assessments will be performed to evaluate delirium outcomes. The research team will also collect relevant information from participant's medical notes if any complications post ICU discharge arise during inpatient and follow-up stages.

3.1. STUDY OUTCOME MEASURES

The study's endpoint, similar to the primary objective of this research study, is to assess the feasibility and acceptability of implementing a nurse-led intervention in ICU, using family-focused sensory stimulation to improve delirium outcomes in patients undergoing cardiac surgery.

This study secondary outcome measures are to develop an innovative nurse-led tool to improve ICU delirium outcomes after cardiac surgery in ICHT and its acceptance amongst patients, family members/friends and critical care staff.

4. PARTICIPANT ENTRY

Recruitment and screening activities will be mainly carried out by the study PI, who is currently funded to support research in the department led by the care team. The PI will aim to work together with the surgical team and when appropriate, add clinicians to the delegation log, such as doctors in clinic. This will allow the supporting clinical team members to refer potential participants to the research team.

4.1. PRE-REGISTRATION EVALUATIONS

Potential eligible participants will be identified by the PI and pre-operative surgical team responsible for their care. All participants will be discussed regularly with the research team to ensure their suitability for the study. Before eligibility is established, PI will remotely screen participant’s electronic medical notes, diagnostics tests and/or attend multidisciplinary team meeting minutes to confirm participants’ suitability to undergo major cardiac surgery.

4.2. INCLUSION CRITERIA

PARTICIPANTS
- Female and male patients over the age of 18
- Participants speaking English language and having mental capacity to consent
- Suitable to undergoing cardiac surgery at ICHT.

FAMILY MEMBERS/FRIENDS
- Nominated by the participant.
- Be willing to record videos and participate in the study.

HEALTHCARE PROFESSIONALS
- Critical Care Nurses that have provided direct care to at least one patient who received the study intervention

4.3. EXCLUSION CRITERIA

PARTICIPANTS
- Female and male patients under the age of 18
- Unable to consent to the study pre-operatively
- Significant hearing/visual impairment
- Participants with learning disabilities, pre-existing delirium, dementia or other significant underlying cognitive morbidity
- Moribund participants, likely to die in the next 24 hours
- Participants that do not speak English language

FAMILY MEMBERS/FRIENDS
- Refuse to consent of gain assent
- Significant hearing/visual impairment

- Family members/friends that do not speak English language

HEALTHCARE PROFESSIONALS
- Critical care nurses that have not been involved in the implementation of the intervention
- Refuse to consent or gain assent
- Significant hearing/visual impairment and non-English speaking

4.4. WITHDRAWAL CRITERIA

PARTICIPANTS WITHDRAWAL CRITERIA

- No longer willing to participate after consent
- Personal consultee wishing to withdraw participants from the study
- Patients that the clinical and/or research team deem too distressed to continue participating in the study
- Participants who have lost capacity to participate in the study after hospital discharge

For participants wishing to not continue in the research study their decision will be respected. Participants will be reassured that their wishes will not affect their care and/or employment in any way. They will also be informed that they are not required to give a reason with regard to not wishing to participate. However, if they are happy, the reason will be recorded in their medical records.

When conducting clinical research, the study team will always respect participants' interests and prioritise them over those of science and society. Therefore, if the participant wishes to be withdrawn from the study, this must be done without delay and appropriately documented in the medical notes. The research team will respect participants' wishes and feelings by ensuring that no study activities are done to and/or carried out in relation to participants who object to continuing in the study. Those participants wishing to withdraw will be asked for permission to keep the data already collected, but if they decline, all data will be destroyed appropriately.

The study team will also assume the professional responsibility to protect participants from the risk of harm and/or prevent them from feeling pain or discomfort. Additionally, the Principal Investigator in agreement with the Chief Investigator will also consider withdrawing any participant who appears to be in too much distress throughout the research study. If so, this will be discussed with the participant and clearly documented on medical notes.

Participants who lose capacity after hospital discharge or die unexpectedly during the course of the study will be automatically withdrawn. If they are withdrawn, any identifiable data already collected with consent will be retained and may be analysed, but no further data will be collected, or any other research procedures carried out in relation to these patients.

4.5. PARTICIPANT IDENTIFICATION AND RECRUITMENT

The identification of potential participants will involve the pre-screening of both electronic and paper notes to confirm their suitability to undergo cardiac surgery. Identification of participants who are suitable for the trial will take place by the PI and the direct care team at the pre-operative clinic in Hammersmith Hospital. The PI will remotely screen from the following recourses: pre-operative cardiac clinic lists, pre-appointment referral lists, cardiac surgical lists

at Imperial College Healthcare NHS Trust and weekly multidisciplinary team meetings. Additional members of the surgical will be added to the delegation log where appropriate, such as doctors working in the clinic. This will allow the supporting team members to refer patients from clinics to the research team.

The following data will be collected for the pre-screening:

- Full Name
- Age
- Hospital number
- Clinic appointment date and time
- Cardiac surgery provisional date and time

Patients and family members/friends will be identified and screened at the pre-operative clinic appointment before the surgery. These patients will be approached face-to-face, following ICHT guidelines and using appropriate personal protective equipment, if necessary. Participants will be given the patient information sheet in person and at least 24 hours to consider their participation in the research study (Annex II, III). Alternatively, potential participants may be contacted via telephone and introduced to the study after being initially approached by the clinical team. Those wishing to take part will be sent an information sheet and consent form via email or in the post (Annex IX). Participants will be asked to nominate a family member/friend and if they would be willing to give a participant information sheet to their chosen person (Annex IV, V). Their family members/friends will be given time to consider their participation and if happy will be consented by the study PI or other trained and delegated members of the study team. Patients will be also ask to select a personal consultee to be involved in situations where they might lack capacity to verbally consent to the study (e.g., delirium episodes). Consultees will be given time to consider their role in the study and will be asked to sign an information sheet (Annex XV, XVI).

Critical care nurses will be identified in ICU after providing direct care to at least one participant enrolled into the research study. They will be required to sign a written consent form (Annex VI, VII) and agree to comply with the study tasks as planned.

The study team will seek written informed consent before enrolling any participant into the study and consent will be dealt with as an ongoing process before any study task takes place. All participants will be then formally discussed with the CI and SIs to ensure their suitability for the study.

4.6. PARTICIPANT CONSENTING

Participants Consent

Study participants (patients, family members/friends and critical care nurses) will be given a full explanation of the study in addition to the participant information sheet. They will then be given as long as they deem necessary to consider their participation in the study. For participants that are approached and happy to consent on the same day, this will be facilitated however all participants will be offered at least 24 hours to decide their participation in the study. Every potential participant will be informed that taking part in this research is voluntary and will not affect their care or employment in any way. They will also be informed they have

the right to refuse, or withdraw at any time, without having to give a reason. If so, this request will be fully respected by the research team.

After consideration if a potential participant wishes take part in the study, they will be taken through an informed consent process where a member of staff on the study delegation log will check their understanding of the study requirements and clarify any possible questions. Once both the researcher and participant are satisfied, written consent will be gained from the participant using a signed paper informed consent form (ICF). This process will be documented in the patient's electronic notes (Cerner), printed and filed in the paper notes along with a copy of the consent form. A copy of the signed paper ICF will be given to all participants enrolling in the study and the original ICF stored in the Investigator Site File (ISF). Based on this consent form obtained at the pre-operative stage, the research team may collect some initial study data from participants, such as:

- Home/personal telephone number
- Email/home address
- Preferred method of contact
- Relevant information in the medical records

Subsequently to the consenting process and considering each participant's preference, the recording/transfer of family videos and digital pictures will be completed at the same appointment as consenting, or during any other hospital appointment arranged within the pre-operative stage. Alternatively, family members have the possibility to self-record the videos at home and send them to the PI via encrypted NHS email. This will be then directly and safely uploaded to individual secured devices, that will be kept in a key-locked cabinet inside the CI's office.

Patients Regaining Consent

Patients undergoing cardiac surgery require mechanical ventilation and sedation to remain in an induced coma throughout the theatre procedure and for a short period of time after being admitted to ICU. Thus, patients' mental capacity is expected to fluctuate during the research study and patients will be unable to promptly re-consent to the study upon ICU admission.

The intra-operative stage is not included in the design for this research study; hence no direct study interventions will be performed on patients during this period. However, the research team may collect any relevant intra-operative information specified in the participant's electronic medical notes, which will happen retrospectively to the operation and written consent for data collection will be sought from participants prior to the procedure.

When appropriate, the study team will re-consent participants in ICU once they are clinically stable, fully awoken from the anaesthesia and have regained the capacity to remember and understand the details of the study. A member of the research team obtaining re-consent will offer to explain the study again and allow time for patients to reconsider their participation in the study. The study will then resume as planned in ICU when participants sign a Patient Regaining Capacity Consent Form (Annex VIII) and express that are happy to proceed with the study intervention in ICU. After written consent is obtained, a copy will be uploaded to medical records (e.g., Cerner), another copy will be given to the patient, and the original

document will be retained in the ISF, which will be stored in a key-locked cabinet inside the CI's office.

In occasions where participants are unable to consent or have lost their capacity to consent more than once whilst in ICU (e.g., delirium), their participation in the study will be considered by approaching their personal consultee. If their participation in the study resumes, videos / photos will be played at the patients' bedside during the planned intervention schedule. Upon delirium diagnosis, the last video played will be shown again to patients with the aim of promoting a reduction in ICU delirium. In the unfortunate eventuality of an urgent clinical deterioration (e.g., urgent induced coma due to mechanical ventilation requirements) that may happen whilst in ICU, their participation in the study will also resume considering their initial consent form signed before surgery and after seeking prior agreement with their personal consultee. In these situations, the Patient Regaining Capacity Consent Form will be collected at a later stage and after patients have re-gained capacity to express their wishes.

For patients who lose mental capacity after hospital discharge, they will be formally discussed with the CI and withdrawn from the research study. The study team will communicate the decision to participants and appropriately document it on medical notes.

Personal Consultees

Personal consultees, who are interested in the patient's welfare but not doing so for remuneration or acting in a professional capacity, will be included in this research study. Consultees can be anyone within the patient's family or friend's group (e.g. relative/friend/partner). When possible, patients will delegate pre-operatively whom they would like to select as personal consultee.

Consultees will be introduced to the study post-operatively and in situations where patients lack capacity to verbalise their wishes (e.g., delirium). They will be given time to consider their role in the study and they will be provided with a study information sheet. If they agree to act as personal consultees for this study, the research team will asked them to consider whether patients would wish to continue taking part in the study or not. When an unanimous decision (between the research team and consultees) has been made, this will be clearly documented on patients' medical notes and consultees will be asked to sign a consent form.

If consultees agree that patients should continue in the study, their participation will resume. However, if personal consultees believe participants should be withdrawn from the study, the research team will respect their decision and make any necessary arrangements.

Notifying the GP

Patients will also be asked to give permission to contact their General Practitioner (GP) and inform them about their participation in this study (Annex X). With the patient's permission and written consent, their GP and other doctors involved in their clinical care will be kept informed of their participation in the study. Otherwise, all patient's information will remain confidential, including anything they say in their interview. We may contact their GP to obtain information about their health status if we cannot reach them. A GP letter will be sent to the GPs of all patients taking part in the study. The GPs of family members/friends and nurses will not require to be notified. All information will remain confidential.

5. ADVERSE EVENTS

Due to the nature of the study design, we do not expect any adverse events or serious adverse events. If a participant should experience an event whilst in attendance of the individual interview, standard hospital pathways will be followed, and documentation will be kept in the patient's medical record and filed in the study site file.

Participation in the study may trigger experiences that patients and family members/friends find it difficult to manage. However, it can also be helpful for participants to discuss their feelings within a supportive environment outside of the care context. Throughout this research study, if any incidental findings and/or other patient's safety concerns are identified by the PI, this will be immediately reported to the CI. Following discussion and in agreement with the CI's decision, any participants' safety concerns will be further discussed with the clinical care team.

6. STUDY ASSESSMENTS

6.1. ASSESSMENT AND FOLLOW UP

STUDY INTERVENTION

The intervention will require family members/friends (selected by patients) to self-record short videos (during the preoperative stage) based on a carefully drafted guided script divided into two parts: a) Reorientation (discussing date/time/surroundings, family members on the video); b) Reassurance (providing comforting/supportive messages). Videos will be recorded either with a video-recorder device (provided by the research team) in the pre-operative clinic or at home using a personal smartphone. At a later stage, videos will be sent to an encrypted NHS email and securely uploaded in line with ICHT Information Governance to a password-protected iPad, which will also facilitate family-patient video calls in ICU. In addition, patients will be requested to select personal pictures pre-operatively, which will be also sent to the PI's encrypted NHS email and securely uploaded into a special digital photo frame with reference to time and date.

Digital photos will be shown continuously at the patient's bedside for 12 hours a day (between 8am to 8pm) and different family videos will be played three times at specific hours of the day (9am, 2pm and 7pm) throughout their stay in ICU. Additionally, videos will be also played upon patient request and last video shown that day will be re-played. In occasions when patients develop ICU delirium and considering personal consultee's agreement, the last video shown to participants will be re-played and patients will be closely monitored. This will help the research team to understand if the combination of personal pictures and family videos impacted patients' orientation and reassurance and if, ultimately, promoted the reduction of ICU delirium. The PI will be responsible for setting up the required equipment and delivering the intervention alongside ICU nurses as illustrated in the study diagram below (Annex XI).

The draft guided script of reorientation and reassurance messages was initially developed based on published research and considering patients' memories from their lived ICU experience(s) [15]. Subsequently, it was further refined within a lay focus group of patient and public members (with ICU lived experience) and reviewed by an expert panel comprising two critical

care consultants, two critical care nurses and one clinical academic research nurse (Annex XIII). Family members and friends will be requested to not deviate from the draft script order of events and to frequently mention the patient's name at the beginning/end of sentences when recording the video. They will be also asked to provide personalised messages in the second part of the video and to use simple terms equivalent to a 5th-grade reading level. The research team expects that part A) of the video to be similar between study participants, but part B) tailored to each individual participant.

Additionally, as previously agreed and supported by the critical care nursing lead for the ICU at Hammersmith Hospital, all critical care nurses will contribute to the study by filling in a daily checklist (Annex XII). This checklist will help the research team to understand the impact of the study intervention and if participants developed ICU delirium. Moreover, as per standard ICU practice at ICHT, patients following cardiac surgery will be closely monitored and primary outcomes regularly collected by critical care nurses.

INPATIENT AND FOLLOW-UP STAGES

Whilst patients remain in ICU, clinical data will be collected from medical records by the PI with the aim of assessing if ICU delirium was developed during this period. Such clinical data is documented and measured routinely as per the standard of care and it includes:

- Relevant medical history
- Relevant intra-op complications
- Vital signs and primary outcomes (e.g., CAM-ICU measurements)
- New prescriptions (associated with prevention/management of ICU delirium)
- Relevant post-operative complications

Subsequently to ICU discharge, each patient will be followed up daily on the wards and one time after hospital discharge at the standard of care monthly follow-up clinic. During this stage, the PI will perform different quality of life questionnaires, such as cognitive and emotional assessments as recommended by the international community when evaluating delirium outcomes in hospital settings [17]. Cognitive and emotional assessments will be performed according to the SoA below and will include the use of the following tools:

- CAM (Confusion Assessment Method)
- Montreal Cognitive Assessment (Annex XVII)
- Generalised Anxiety Disorder Assessment-7 (Annex XVIII)
- Patient Health Questionnaire-9 (Annex XIX).

Any relevant clinical complication that might arise during the follow-up period, will be also collected by the PI and further discussed with the CI and SI.

INTERVIEWS

The interviews will be made in accordance with the study participants' schedule and will run for approximately 20 minutes.
Two dyadic interviews with patients and family members/friends will take place at the hospital during the inpatient stay time: the first will be held on the day of ICU discharge and the second on the day of hospital discharge. The third interview will happen at the post-operative follow-

up appointment or virtually (depending on participants' preference) one month after hospital discharge. Where possible, family members/friends and patients interviews will be conducted together, but the possibility of performing interviews separately will be given, so that participants feel able to discuss opinions freely without influence. Interviews directed to healthcare professionals will take place either virtually or in private rooms located in ICU, considering physicians' availability and preferences. In such circumstances, researchers will follow local Trust guidance on the use of personal protective equipment and social distancing. All interviews will be conducted by the PI. They will be audio-recorded and transcribed by the PI and the research supervisors. An ICHT encrypted recorder will be used for this process. All data will be anonymised and stored according to the ICHT guidance.

6.2. END OF STUDY

The end of the study is defined as the last subject study visit, including follow-up period. Data from this study will be kept confidential at all times. Only staff on the delegation log will have access to the study data. No identifiable data will be published or made public during this study. The Sponsor (ICHT) will store identifiable information and study documentation about the participants for a minimum of 10 years after the completion of the study.

7. STATISTICS AND DATA ANALYSIS

The study sample size was based on sample size based on previous feasibility studies and complexity of the planned intervention [18]. Considering the study design and timelines, the recruitment target for this study is 12 patients alongside 12 family members. The research team also plans to recruit 6 critical care nurses to be able to collect more in-depth data about the study intervention acceptance in ICU.

Quantitative data will be analysed using SPSS statistical software. Descriptive statistics will be used to present demographic data, medical characteristics and causal beliefs about ICU delirium. Correlations will be used to examine the relationships between vital signs, new ICU prescriptions, ICU complications, mortality rates and delirium status. Logistic regression analyses will be applied when investigating the influence of sociodemographic and clinical factors on the identification of patients with ICU delirium.

Qualitative data from interviews will be audio-recorded, transcribed verbatim and managed in NVIVO. Framework analysis will be used to systematically manage and holistically interpret qualitative data collected from the interviews. The academic supervisors, Professor Stephen Brett, Professor Natalie Pattison and the CI, Dr. Sanooj Soni, will be involved in reviewing transcripts, developing the coding frame and identifying potential themes for subsequent exploration. Additionally, the PI will compare concepts from existing literature with those emerging from this study. After identifying the initial themes, the lead researcher will also liaise with a patient and public involvement (PPI) advisory group to check the credibility and usefulness of the selected themes and findings.

8. REGULATORY ISSUES

8.1. ETHICS APPROVAL

The Study Coordination Centre has obtained approval from the Bradford and Leeds Research Ethics Committee (REC) and Health Research Authority (HRA). The study must also receive confirmation of capacity and capability from each participating NHS Trust before accepting participants into the study or any research activity is carried out. The study will be conducted in accordance with the recommendations for physicians involved in research on human subjects adopted by the 18th World Medical Assembly, Helsinki 1964 and later revisions.

8.2. CONSENT

<u>Participants Able to Consent</u>

Consent to enter the study will be sought from each participant (patients, family members/friends and critical care nurses) only after a full explanation has been given, an information leaflet offered, and time allowed for consideration. A signed participant consent must be obtained before their enrolment into the study. The right of the participant to refuse to participate without giving reasons must be respected by the research team. All participants are free to withdraw at any time from the protocol treatment without giving reasons and without prejudicing further treatment. They will be made aware that their participation in the study will not affect their care or employment in any way. The study team will deal with consenting as an ongoing process and, before any study tasks take place, participants will be asked if they are happy to remain in the study. The research team will also ensure that participant's wishes are documented on medical notes and the clinical team is aware of the study team's plan of action.

<u>Participants Unable to Consent</u>

A participant is deemed unable to decide whether to take part in research if they cannot:

- Understand the information relevant to the decision (information should be given in a way that is appropriate to the particular participant)
- Retain that information for long enough to conclude the decision (this may be for a relatively short time, but still long enough to enable decision-making to occur)
- Use or weigh that information as part of the process of making the decision (participants need to understand the consequences of each option).
- Communicate their decision (whether by talking, using sign language or any other means).

Due to the specificity of the care group, patient's mental capacity is expected to fluctuate throughout the study and if so, a personal consultee will be included to decide whether patients should continue taking part in the research study. A Patient Regaining Capacity Consent Form should be signed post-operatively when possible, ideally before participants resume with the study in ICU, and after periods of lacking capacity.

Decisions weather or not the participants have the capacity to give consent will be made by the PI or a Senior Researcher, who has appropriate training and experience in assessing consent. The PI has been formally trained on how to obtain informed consent from participants and aware of the Mental Capacity Act 2005.

8.3. CONFIDENTIALITY

The CI and all members of the delegated study team will preserve the confidentiality of participants taking part in the study. All data will be collected and managed in line with the Data Protection Act 2018 and GDPR.

The risk of confidentiality breach will be minimised by ensuring all study-related data is stored securely in a pseudonymised form and participants will be identified by a study ID number only. Any patient-identifiable data that is shared with parties outside of the research study team will be modified such that it is in a linked anonymised form (with removal of patient hospital number, NHS number, name, date of birth, etc.). Additionally, the research team will ensure that any patient-identifiable data is safely kept in a key-locked cabinet only accessible to members of the study team and remaining study documentation is always securely stored in a password protected computer and/or research office.

8.4. DATA STORAGE AND MANAGEMENT OF DATA

The research team will follow the principles of Good Clinical Practice (GCP), the EU GDPR and UK Data Protection Act 2018 to ensure all the data collected will be anonymised and the confidentiality of all participants will be protected. We do not anticipate any safety concerns associated with this study, for either participants or the researchers involved.

Patient demographics and meta-data will be recorded for all patients. All pre- and post-screened data collected will be kept on an ICHT NHS computer and be password protected. Only members of the study delegation log will have access to these electronic files. Original consent forms will be kept in the ISF and one copy will be uploaded in the patients' electronic notes. The ISF will be kept with the CI, in a key-locked cabinet inside a key/password-locked office accessible only to members of the research team who have signed the delegation log.

This study involves the collection of questionnaires, participant's pictures and the use of audio-visual recording devices with patients, family members/friends and healthcare professionals, which requires ethical approval. All responses to questionnaires and interviews will be pseudonymised to ensure confidentiality. This will be done by assigning a study ID number to all participants enrolling into the research study, which will be used for the questionnaires and to address participants during the interview. The use of a study ID number will then be used throughout the study and data analysis. The research team will also create single participant's study folders, which will contain individualised anonymised data collected during the study (e.g., questionnaires, nurses' checklists, etc.). Participant's folders will be retained in the key-protected research offices, only accessible by members of the study team. Additionally, the study team will delete participants contact details after concluding the study data collection and results shared with consented participants.

Interviews will be recorded with an encrypted audio-recording device provided by ICHT and videos with a study video-camera that will be kept in a key locked cabinet inside the CI's office. All interview data and electronic transcripts (when transcribed by a member of the research team) will be stored securely on an encrypted and secure institutional password protected ICHT computer. Interview recordings will be deleted as soon as the transcription has been completed and analysed by the research team. Video will be recorded either with a video-recorder device that belongs to the study team or self-recorded by family member/friends at home.

Subsequently, videos and pictures will be sent to the PI's encrypted NHS email and then safely uploaded to a study password protected iPad and into a digital photo frame, respectively. All video recordings and pictures will be deleted from the devices as soon as the individual participant's intervention is finished and at ICU discharge. All videos stored in the study team's video-recorded device will be deleted after the intervention is finished or upon ICU discharge. No family video and/or personal pictures digital copies will be retained by the study team without participant's prior consent.

All published data will be pseudonymised, including any direct quotations we may include to illustrate key themes. Files containing any identifiable information will only be available to the PI and appropriate members of the study team on the delegation log. These files will be password-protected and kept separately from files where only the unique study ID is used.

Study closure

Following data analysis, dissemination and study closure, the research team will retain essential documents until notified by the Sponsor (ICHT) and then archived for at least ten years after study completion, as per ICHT policy. Participant's study files and other source data (including copies of protocol, PIS, records of informed consent, and other documents pertaining to the conduct of the study) must be kept for the maximum period of time permitted by the institution.

Documents should be stored in such a way that they can be accessed/data retrieved at a later date. Consideration should be given to security and environmental risks. No study document will be destroyed without prior written agreement between the Sponsor and the research team. Should the research team wish to assign the study records to another party or move them to another location, a prior written agreement must be obtained from the Sponsor.

Reducing bias

All interviews will be carried out by the same researcher throughout the study to reduce differences in the way questions are asked and will be audio recorded and transcribed verbatim prior to analysis. Recruitment of participants and analysis will be carried out simultaneously allowing for constant comparison and the identification of similarities. Any data gathered will be then analysed using a framework approach.

It is essential to acknowledge that the PI will be carrying out interviews and is currently a supporting member of the direct care team. The PI has access to patient records through various electronic systems and will be using these records as a means to pre-screen prior to the approaching and enrolment of potential participants. Although clinicians can very effectively use transferable skills from clinical interviewing to interviewing for research purposes, the blurring of the two roles could potentially present an ethical bias. While the primary researcher is conducting this study, the PI will assume the responsibility to not provide direct care to any participants involved in the project. The PI will also aim to be as transparent as possible regarding the clinical position as a critical care researcher, throughout the study process, including interviewing, analysis of the results and dissemination of the findings. Data analysis will be conducted by the primary researcher, and emerging themes will be further discussed with the CI and SIs.

8.5. INDEMNITY

The Sponsor, Imperial College Healthcare NHS Trust, holds a standard NHS hospital indemnity and insurance cover with NHS Resolution for NHS Trusts in England, which apply to this study.

8.6. SPONSOR

Imperial College Healthcare NHS Trust (ICHT) will act as the main Sponsor for this study. Delegated responsibilities will be assigned to the NHS trusts taking part in this study.

8.7. FUNDING

This research study is being undertaken as part of a Pre-Doctoral Research Fellowship that has been awarded to the PI, Maria Reguenga, by the IHC and is being funded by the NIHR Imperial BRC.

We currently do not have funding to support the participation of patient, family members and healthcare professionals. Additionally, other members of the research team will not receive any money contributions from supporting this study.

8.8. AUDITS

The study may be subject to audit by ICHT under their remit as sponsor and other regulatory bodies to ensure adherence to GCP and the UK Policy Framework for Health and Social Care Research. The Sponsor may also carry out audits to ensure compliance with the protocol and appropriate local regulations.

Direct access will be granted to authorised representatives from the Sponsor/Host institution (ICHT), the partner institution (Imperial College of London) and the regulatory authorities to permit trial-related monitoring, audits and inspections.

9. STUDY MANAGEMENT

Daily Study Management

The day-to-day management of the study will be co-ordinated from Hammersmith Hospital by the PI. The CI will assume the responsibility to supervise the PI throughout the study and discussion about the study management will occur during weekly meetings. Study data management and analysis will be also discussed with SIs at a later stage of the research study.

Protocol Amendments

Every care was taken when drafting this study protocol, but corrections or amendments may be necessary. These will be circulated to investigators in the study prior to re-submission. Problems relating to this study should be referred, in the first instance, to the Chief Investigator

No amendments to this protocol will be made without prior consultation and agreement with the Sponsor. Any amendments to the study that appear necessary during the study must be

discussed with the Research Team and the Sponsor concurrently. If agreement is reached concerning the need for an amendment, it will be produced in writing by the PI and will be made a formal part of the protocol following ethical and regulatory approval.

The research team is responsible for ensuring that any substantial amendments to an approved study protocol, are not to be initiated without previous NHS REC review/approval and not until the REC's approval letter has been obtained by the research team.

Protocol Deviations

Any protocol deviations will be documented in a protocol deviation form and safely filed in the ISF. The CI and SIs will be made aware of the protocol deviation. Corrective and preventative actions will be discussed and implemented to prevent new deviations to the study protocol.

Study Reports

Study outcomes will be regularly reported by the PI to the CI and SI and further discussed during monthly meetings. Two different reports will be sent to the Imperial Health Charity to declare any study mid-year and end of study progress with the aim to ensure their continuum support in the research study.

The Sponsor and REC will be also sent an annual/end of study report upon request and will be informed about study closure, within the required timelines.

10. PUBLICATION POLICY

The PI will co-ordinate dissemination of data from this study and ensure all published/presented data will be pseudonymised. All publications (e.g., manuscripts, abstracts, oral/slide presentations, book chapters) based on this study will be reviewed by each sub-investigator prior to submission. All communication concerning the study, including at a conference or seminar, shall acknowledge the responsible parties and the invaluable Imperial Health Charity financial contribution to the study.

11. REFERENCES

[1] Lange P. J Clin Nurs. 2019 Jul; 28(13–14):2537-2542.
[2] Mart M. Semin Respir Crit Care Med. 2021 Feb; 42(1):112–126.
[3] Lynch J. Aust Crit Care. 2020 Sep; 33(5):475–479.
[4] MacLullich A. Health Technol Assess. 2019Aug; 23(40):1-194.
[5] Schmitt, E. The Gerontologist vol. 59,2 (2019): 327-337.
[6] Liang S. Intensive Crit Care Nurs. 2022 Dec; 103369.
[7] Brown CH. Curr Opin Anaesthesiol. 2014 Apr; 27(2):117–122.
[8] Pagad S. Cureus. 2020 Aug; 12(8):e10096.
[9] Evans A. Ann Card Anaesth. 2016 Apr-Jun; 19(2):328-37.
[10] Crown. Intensive Care Top James Lind Alliance: Priority Setting Partnership. Weblog. https://www.jla.nihr.ac.uk/priority-setting-partnerships/intensive-care/top-10-priorities/ (Accessed 10th January 2023).

[11] Habeeb-Allah A. Nurs Crit Care. 2021 May; 26(3):150–155.
[12] National Institute for Health and Care Excellence (NICE). THINK delirium in intensive care. Weblog. https://www.nice.org.uk/sharedlearning/think-delirium-in-intensive-care (Accessed 5th September 2023).
[13] Qin M. J Clin Neurosci. 2022 Feb; 96:114–119.
[14] Deng L. J Crit Care. 2020 Dec; 60:241-248.
[15] Munro C. Heart Lung. 2017 Jul-Aug; 46(4):234-238.
[16] Hung L. Contemp Nurs. 2018 Aug-Oct; 54(4-5):350-361.
[17] Rose L. Crit Care Med. 2021 Sep; 49(9):1535-1546.
[18] Lewis M. Pilot Feasibility Stud. 2021 Feb; 7(1):40.
[19] Larner, AJ. Cognit Scr Instru: A Practical Approach – 2nd Ed. 2017; pp.139–195. ISBN 978-3-319-44774-2
[20] Nasreddine, Z. MoCA Test. Weblog. https://mocacognition.com/ (Accessed 7th December 2023).
[21] Johnson, Sverre Urnes et al. Front in Psyc. 2019 Aug; vol.10:1713.
[22] Kroenke, K et al. J of Gen Int Med. 2001 Sep; 16(9):606-13.

APPENDICES/ANNEXES

- ANNEX I – Schedule of Assessments (SoA)
- ANNEX II – PIS for Patients
- ANNEX III – ICF for Patients
- ANNEX IV – PIS for Family Members/Friends
- ANNEX V – ICF for Family Members/Friends
- ANNEX VI – PIS for Critical Care Nurses
- ANNEX VII – ICF for Critical Care Nurses
- ANNEX VIII – ICF for Patient Regaining Capacity Consent Form
- ANNEX IX – Draft Invitation Email to Participants, Family Members/Friends and Nurses
- ANNEX X – GP Letter for Participants
- ANNEX XI – Participant's pre-operative pathway and intervention delivery diagram
- ANNEX XII – Critical Care Nurse's checklist
- ANNEX XIII – Video Guided Script Template for Family Members/Friends
- ANNEX XIV – Participant's post-ICU discharge and follow-up pathway
- ANNEX XV - PIS for Personal Consultees
- ANNEX XVI – ICF for Personal Consultees
- ANNEX XVII - Montreal Cognitive Assessment Questionnaire
- ANNEX XVIII - Generalised Anxiety Disorder Assessment-7
- ANNEX XIX - Patient Health Questionnaire-9

ANNEX I - Schedule of Assessments (SoA)

Exam	Pre-treatment (Clinic)	Treatment (ICU)				Inpatient follow-up (Ward)	Outpatient follow-up (Clinic)
**Study Days**	-D60 to D0	D0	D1	D2	D3	D4, D5, D6, D7, D8, D9, D10	D30
Informed Consent	X						
Cardiac surgery		X					
ICU Family Intervention			X	X	X		
CAM-ICU and RASS		X	X	X	X		
Vital Signs (Heart Rate, Blood Pressure, Respiratory Rate, Temperature, Saturations)	X[1]	X[1]	X[1]	X[1]	X[1]	X[1]	X[1]
Dyadic Interviews					X[2]	X[2]	X[2]
Nursing interviews					X[3]		
Montreal Cognitive Assessment						X[4]	X[4]
Generalised Anxiety Disorder Questionnaire-7						X[4]	X[4]
Patient Health Questionnaire-9						X[4]	X[4]
CAM						X[5]	X[5]

[1]Vital signs to be collected at least one time during pre-treatment, inpatient follow-up, and outpatient follow-up stages; and daily highest/lowest vital signs during ICU stay.
[2]Participants and family members/friends will be approached for dyadic interviews three times: day of ICU discharge, day of hospital discharge and up to one month post ICU discharge to discuss the intervention and assess delirium complications. Whether possible, interviews will be performed together, but single interview option will be provided.
[3]Critical care nurses will be approached for one single interview to evaluate intervention acceptability, useability, and possible study limitations.
[4]All quality-of-life questionnaires (QoL) are to be performed by participants one time during the inpatient stage and another time at the outpatient follow-up clinic appointment.
[5]Short-term delirium outcomes post ICU discharge will be evaluated by performing daily CAM assessments during the inpatient period and once at the follow-up appointment.

ANNEX XI - Participant's pre-operative pathway and intervention delivery diagram

ICHT Pre-Operative Clinic

Recruitment Patients/Relatives

Selection of potential eligible patients in ICHT pre-op cardiac surgery clinic

Clinic Pre-Op Appointment 1 – Explain research study and provide participant information sheet

Clinic Pre-Op Appointment 2 – Patient and family/friend sign informed consent form; Clarification of any questions; Patient provides selected pictures; Recording of family videos.

Equipment set up – Securely upload photos to digital photo frame and video to the iPad

ICHT Cardiac Intensive Care Unit

ICU Admission post cardiac surgery (Day 0, ~ 1pm)

Patient stable and fully awake after extubation (Day 0, ~ 3pm)

Principal Investigator: Re-consents participants to the study (if possible); Delivers the study intervention by turning on the digital photo frame device, showing pre-selected pictures, and playing first family video on the iPad in ICU (Day 0, ~6pm)

Digital photos will be continuously shown between 8am and 8pm.

+

Family videos will be delivered whilst participants are in ICU:
- Three times daily (PI to deliver videos at 9am and 2pm and ICU nurses at 7pm).
- Upon participants' request (the last video will be re-played)
- When participants show signs of delirium (the last video will be re-played) and patients will be closely monitored.

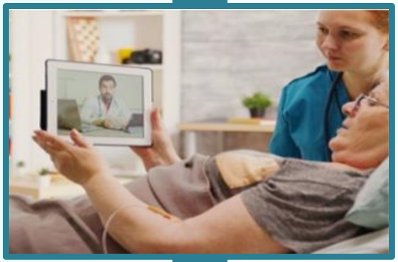

ICU discharge (~ Day 2/3) and first dyadic interview

ANNEX XII – Critical Care Nurse's checklist

Table 1. Nursing Checklist Daytime

N	Nursing Checklist Daytime (8am – 8pm)	Tick as appropriate
1	Did the patient have any unknown visual/auditory impairment?	☐ Yes ☐ No
2	Were any visual/auditory aids (eg. glasses/hearing aids) in place during the intervention delivery?	☐ Yes ☐ No ☐ N/A
3	Was the family intervention delivered 3 times during the day (8am-8pm)?	☐ Yes ☐ No<br>Times: 1. ____:____; 2.____:____;<br>3. ____:____
4	Did the patient express any verbal/non-verbal emotions when watching the video?	☐ Yes ☐ No<br>If "Yes", please specify: _____
5	Did the patient look at the digital photo frame during the day?	☐ Yes ☐ No<br>If "Yes", how many times: _____
6	Did the intervention impact ICU nursing care?	☐ Yes ☐ No<br>If "Yes", please specify: _____
7	Was the family intervention delivered without any untoward events (eg., acute distress)?	☐ Yes ☐ No<br>If "No", please specify: _____
8	Did the patient request to watch the video more than 3 times a day?	☐ Yes ☐ No<br>If "Yes", please specify the total number of times: _____
9	Did the patient develop delirium during the day (8am-8pm)?	☐ Yes ☐ No<br>9a. If "Yes", was the video played again?<br>☐ Yes ☐ No<br>9b. If "Yes", did it improve delirium status?<br>☐ Yes ☐ No

Table 2. Nursing Checklist Night-time

N	Nursing Checklist Night-time (8pm – 8am)	Tick as appropriate
1	Were any visual/auditory aids removed prior to sleep?	☐ Yes ☐ No
2	From a nurse's perspective, how many hours did the patient sleep?	_______ hours
3	Did the patient request to watch the video overnight?	☐ Yes ☐ No<br>If "Yes", how many times: _______
4	Did the patient develop delirium overnight?	☐ Yes ☐ No<br>4a. If "Yes", was the video played again?<br>☐ Yes ☐ No<br>4b. If "Yes", did it improve delirium status?<br>☐ Yes ☐ No
5	Was the iPad plugged in to charge overnight?	☐ Yes ☐ No

ANNEX XIII - Video Guided Script Template for Family Members/Friends

VIDEO GUIDED SCRIPT TEMPLATE FOR FAMILY MEMBERS/FRIENDS

This draft script of reorientation and reassurance messages was initially developed based on published research and considering patients' memories from their lived ICU experience(s). Subsequently, it was further refined through coproduction within a lay focus group of patient and public members (with ICU lived experience) and reviewed by an expert panel comprising two critical care consultants, two critical care nurses and one clinical academic research nurse.

The template below highlights what will need to be covered during the 3-minute videos and in which order of events this would be ideal to be recorded by participants. This script will assist family members/friends to follow a clear and structured speech, as well as, allow them to better prepare for the video recording process.

Family members and friends will be requested to not deviate from the draft script order of events and to frequently mention the patient's name at the beginning/end of sentences when recording the video. They will be also asked to provide personalised messages in the second part of the video and to use simple terms equivalent to a 5th-grade reading level.

By following this script, the research team expects that part A) of the video to be similar between study participants, but part B) tailored to each individual participant. Videos will be divided into two main parts:

A) Reorientation

- Start the video by greeting the patient and mentioning their name.

- Introduce yourself (e.g., name/preferred name) and your relationship to the patient.

- State where you are (e.g., house, garden, etc.) and if you are alone or accompanied (e.g., with significant others, pets, special objects/toys)

- Mention the patient's name in the beginning/end of the next sentences

- Provide a reference to the approximate time (9am, 2pm or 6pm) and date (e.g., Wednesday)

- Mention that patients have now had their surgery and are staying in Intensive Care at Hammersmith Hospital in London.

- State how many days have been since surgery (e.g., today is day 1 after your cardiac surgery)

B) Reassurance

- Continue the video by explaining that this is a recorded video/message to help patients understand what is going on around them.

- Family members should then choose from the following options but are also free to personalise the next one or two final sentences of the video.

Examples of reassurance and supportive messages:

1) It is OK, do not be scared.

2) You will soon be home with us.
3) Your nurses and doctors are here looking after you and helping you recover.
4) You might be uncomfortable or in pain, but the nurses are making you more comfortable and giving you painkillers.
5) It is loud and noisy because of the machines that are helping you get better.
6) You may have some wires and tubes in place to help you recover.
7) You may have something on your wrists to keep you from pulling at the wires and tubes by accident, but this will soon be removed.
8) You can’t talk right now because of your breathing tube, but the nurses know you might be uncomfortable and are giving you medicine for that.

- Finish the video with a farewell message (e.g., see you soon) and mention the patient's name at the beginning/end of the sentence.

Annex XIV – Participant's post-ICU discharge and follow-up pathway

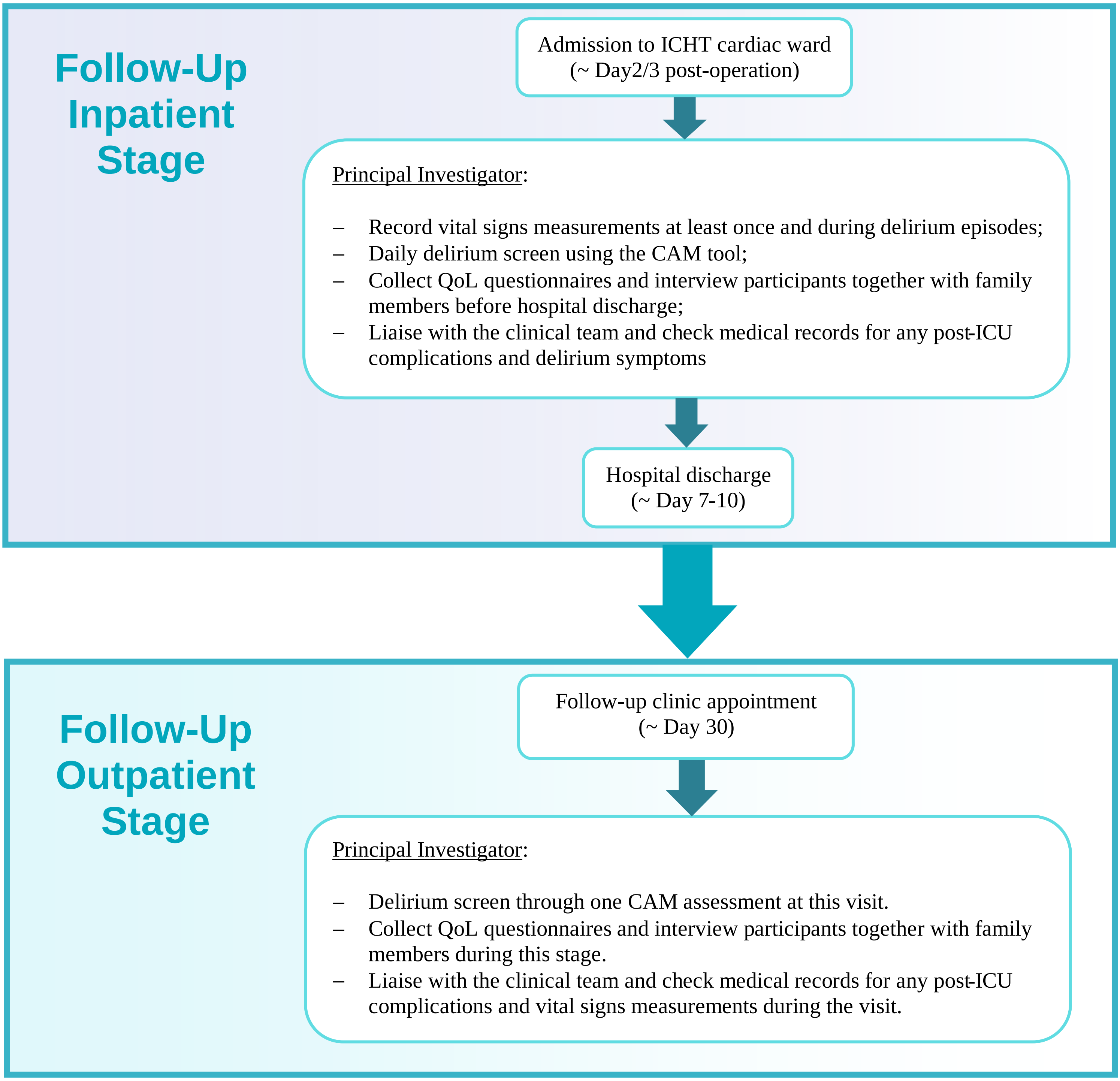

Annex XVII - Montreal Cognitive Assessment (MoCA) Questionnaire

MONTREAL COGNITIVE ASSESSMENT (MoCA®)

Name:
Education:
Sex:
Date of birth:
DATE:

VISUOSPATIAL / EXECUTIVE

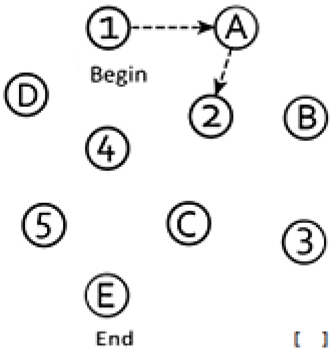

[ ]

Copy bed

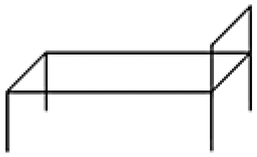

[ ]

Draw CLOCK (Five past ten)
(3 points)

[ ] Contour [ ] Numbers [ ] Hands

POINTS: __/5

NAMING

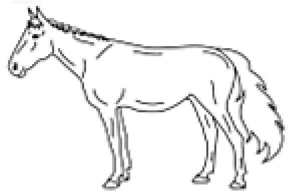

[ ]

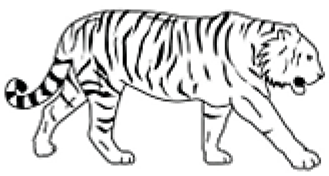

[ ]

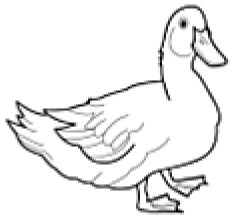

[ ]

__/3

MEMORY

Read list of words, subject must repeat them. Do 2 trials, even if 1st trial is successful. Do a recall after 5 minutes.

	LEG	COTTON	SCHOOL	TOMATO	WHITE
1st TRIAL					
2nd TRIAL					

NO POINTS

ATTENTION

Read list of digits (1 digit/sec.). Subject has to repeat them in the forward order. [ ] 2 4 8 1 5
Subject has to repeat them in the backward order. [ ] 4 2 7 __/2

Read list of letters. The subject must tap with his hand at each letter A. No points if ≥ 2 errors.
[ ] F B A C M N A A J K L B A F A K D E A A A J A M O F A A B __/1

Serial 7 subtraction starting at 60. [ ] 53 [ ] 46 [ ] 39 [ ] 32 [ ] 25
4 or 5 correct subtractions: **3 pts**, 2 or 3 correct: **2 pts**, 1 correct: **1 pt**, 0 correct: **0 pt** __/3

LANGUAGE

Repeat: The child walked his dog in the park after midnight. [ ]
The artist finished his painting at the right moment for the exhibition. [ ] __/2

Language Fluency. Name maximum number of words in one minute that begin with the letter B. [ ] ____ (N ≥ 11 words) __/1

ABSTRACTION

Similarity between e.g. banana - orange = fruit [ ] hammer - screwdriver [ ] matches - lamp __/2

DELAYED RECALL

Memory Index Score (MIS)	(MIS)		LEG	COTTON	SCHOOL	TOMATO	WHITE		
	X3	Has to recall words WITH NO CUE	[ ]	[ ]	[ ]	[ ]	[ ]	Points for UNCUED recall only	__/5
	X2	Category cue						MIS = ____/15	
	X1	Multiple choice cue							

ORIENTATION

[ ] Date [ ] Month [ ] Year [ ] Day [ ] Place [ ] City __/6

TOTAL __/30

MoCA Scoring: abnormal ≤ 25, normal > 25 [19,20].

Annex XVIII - Generalised Anxiety Disorder Questionnaire -7 (GAD-7)

Over the last two weeks, how often have you been bothered by any of the following problems?	**Not at all**	**Several days**	**More than half the days**	**Nearly every day**
1. Feeling nervous, anxious, or on edge	0	1	2	3
2. Not being able to stop or control worrying	0	1	2	3
3. Worrying too much about different things	0	1	2	3
4. Trouble relaxing	0	1	2	3
5. Being so restless that it is hard to sit still	0	1	2	3
6. Becoming easily annoyed or irritable	0	1	2	3
7. Feeling afraid, as if something awful might happen	0	1	2	3
Total Score	______ =	______ +	______ +	______

**If you checked off any problems, how difficult have these problems made it for you to do work, take care of things at home, or get along with other people?**			
Not difficult at all ☐	Somewhat difficult ☐	Very difficult ☐	Extremely difficult ☐

GAD-7 Anxiety Severity Scoring: 0-4 minimal anxiety, 5-9 mild anxiety, 10-14 moderate anxiety, 15-21 severe anxiety [21].

Annex XIX - Patient Health Questionnaire-9

Over the last two weeks, how often have you been bothered by any of the following problems?	**Not at all**	**Several days**	**More than half the days**	**Nearly every day**
Little interest or pleasure in doing things?	0	1	2	3
Feeling down, depressed, or hopeless?	0	1	2	3
Trouble falling or staying asleep, or sleeping too much	0	1	2	3
Feeling tired or having little energy?	0	1	2	3
Poor appetite or overeating?	0	1	2	3
Feeling bad about yourself - or that you are a failure or have let yourself or your family down?	0	1	2	3
Trouble concentrating on things, such as reading the newspaper or watching television?	0	1	2	3
Moving or speaking so slowly that other people could have noticed? Or the opposite - being so fidgety or restless that you have been moving around a lot more than usual?	0	1	2	3
Thoughts that you would be better off dead, or of hurting yourself in some way?	0	1	2	3
Total Score	______ =	______ +	______ +	______

**If you checked off any problems, how difficult have these problems made it for you to do work, take care of things at home, or get along with other people?**			
Not difficult at all<br>☐	Somewhat difficult<br>☐	Very difficult<br>☐	Extremely difficult<br>☐

Depression Severity Scoring: 0-4 none, 5-9 mild, 10-14 moderate, 15-19 moderately severe, 20-27 severe [22].
